# Decomposition methods for the two-stage stochastic Steiner tree problem

**DOI:** 10.1007/s10589-017-9966-x

**Published:** 2017-11-20

**Authors:** Markus Leitner, Ivana Ljubić, Martin Luipersbeck, Markus Sinnl

**Affiliations:** 1 University of Vienna, Department of Statistics and Operations Research, Faculty of Business, Economics and Statistics, Vienna, Austria; 2 ESSEC Business School of Paris, Cergy-Pontoise, France; 3 INOCS, INRIA Lille-Nord Europe, Villeneuve d’Ascq, France

**Keywords:** Lagrangian relaxation, Benders decomposition, Stochastic optimization, Steiner trees

## Abstract

A new algorithmic approach for solving the stochastic Steiner tree problem based on three procedures for computing lower bounds (dual ascent, Lagrangian relaxation, Benders decomposition) is introduced. Our method is derived from a new integer linear programming formulation, which is shown to be strongest among all known formulations. The resulting method, which relies on an interplay of the dual information retrieved from the respective dual procedures, computes upper and lower bounds and combines them with several rules for fixing variables in order to decrease the size of problem instances. The effectiveness of our method is compared in an extensive computational study with the state-of-the-art exact approach, which employs a Benders decomposition based on two-stage branch-and-cut, and a genetic algorithm introduced during the DIMACS implementation challenge on Steiner trees. Our results indicate that the presented method significantly outperforms existing ones, both on benchmark instances from literature, as well as on large-scale telecommunication networks.

## Introduction

The two-stage stochastic Steiner tree problem with complete recourse (SSTP) is a generalization of the well-studied (deterministic) Steiner tree problem (STP), with applications in telecommunication network design under uncertainty. The problem has been introduced by Gupta et al. [14] and subsequently, algorithms based on fixed-parameter tractability [23], heuristics [19], and exact methods [2, 27] have been proposed. Moreover, approximation algorithms for several variants of the problem have been studied [15–17, 36].

Recall that in the classical (deterministic) STP on graphs, one is given an edge-weighted graph with a set of *terminals* that need to be connected at minimum cost (see, e.g., [20]). As illustrated in [27], in the SSTP (and in stochastic network design problems, in general), both the set of terminals and the edge-weights can be subject to uncertainty. In that case, network planners want to establish profitable connections now (in the *first stage*) while taking possible uncertain outcomes into account. Usually, the set of uncertain outcomes is approximated through a set of possible *scenarios*, with a known probability of occurrence. In the *second stage*, the actual scenario is revealed (i.e., the set of terminals and edge-weights become known), and additional connections can be purchased (through so-called *recourse* actions) to create a feasible Steiner tree. The objective is to minimize the *expected cost* of the solution, i.e., the sum of the first-stage cost plus the expected cost of the second stage.

The SSTP is formally defined as follows.

### Definition 1

(*Stochastic Steiner tree problem (SSTP)*) Let $G=(V,E)$ be an undirected graph with root node $r\in V$, first-stage edge costs $c^0\,{:}\,E \mapsto \mathbb {R}_{\ge 0}$ and scenario set *K*. Each scenario $k \in K$ has probability $p^k\in (0,1], \sum _{k\in K} p^k = 1$, as well as second-stage edge costs $c^k\,{:}\,E \mapsto \mathbb {R}_{\ge 0}$ and terminals $T^k\subseteq V, r \in T^k$. The objective is to select first-stage edges $E_S^0\subseteq E$ and second-stage edges $E_S^k\subseteq E$ for each $k\in K$ such that the subgraph induced by $E_S^0\cup E_S^k, G[E_S^0\cup E_S^k]$, connects $T^k$ and the expected cost

$$\begin{aligned} \sum _{e \in E_S^0} c^0_e + \sum _{k \in K} p^k \sum _{e \in E_S^k} c^k_e \end{aligned}$$

is minimized.

*Our contribution*

For the deterministic STP a wealth of theoretical results [6, 11, 20, 29] and empirically successful computational techniques are known [8, 10, 31]. However, as noted in [2, 27], the generalization of results from the STP to the SSTP is not straightforward. In this article we first provide a new integer linear programming (ILP) formulation for the SSTP and show that it is the strongest (in terms of the quality of linear relaxation bounds) among existing formulations. Moreover, we show how the new formulation allows the simple derivation of procedures for computing lower bounds. Overall, we study three such procedures for the SSTP, namely dual ascent, Lagrangian relaxation, and Benders decomposition. The dual information provided by each of these methods is exploited in a common algorithmic framework. This results in a powerful primal–dual method in which the calculation of upper and lower bounds is combined with variable fixing for decreasing the size of the search space.

The effectiveness of our method is demonstrated in an extensive computational study on benchmark instances from the literature, and on large-scale telecommunication networks. We compare our method with the state-of-the-art exact approach from [2, 27], which employs a Benders decomposition based on two-stage branch-and-cut (B&C), and a genetic algorithm from [19], introduced during the DIMACS implementation challenge on Steiner trees. Our results indicate that the presented method significantly outperforms the alternative approaches from the literature, both in terms of computing times, and the quality of obtained solutions.

*Outline*

In the remainder of this section, notation is introduced and related work is discussed. In Sect. 2, a new ILP formulation for the SSTP is presented and its strength is compared to the previously strongest formulation. Moreover, strengthening inequalities are analyzed. In Sect. 3, an algorithmic framework is described which combines a dual ascent procedure, a Lagrangian heuristic, Benders decomposition, and variable fixing. In Sect. 4, computational results are presented, while concluding remarks are drawn in Sect. 5.

*Notation*

Let $G_D=(V,A)$ denote the bidirected counterpart of $G=(V,E)$, where $A=\{(i,j)\,{:}\,\{i,j\}\in E\}$. We leave the arc costs on *A* unchanged, i.e., for all $(i,j) \in A$ and $k \in K$, we have: $c^0_{ij} = c^0_e$ and $c^k_{ij} = c^k_e$, where $e=\{i,j\} \in E$. For $W\subset V$, let $\delta ^+(W) := \{(i,j)\in A : i\in W,j\in V{\setminus } W \}$ be the *outgoing* arc set, $\delta ^-(W) := \{(i,j)\in A\,{:}\,i\in V {\setminus } W,j\in W \}$ the *ingoing* arc set, and $\delta (W) := \{\{i,j\}\in E\,{:}\,i\in V {\setminus } W,j\in W \}$ the undirected cut set. For brevity, if $W=\{i\}$, we write $\delta ^+(i), \delta ^-(i)$, and $\delta (i)$, respectively. For each $k \in K$, let $\mathcal {W}^k$ be the family of node sets inducing *Steiner cuts* with respect to the set of terminals $T^k$, i.e.,

$$\begin{aligned} \mathcal {W}^k:=\{W \subset V\,{:}\,r \notin W, W \cap T^k\ne \emptyset \}. \end{aligned}$$

For a given $k \in K$ and $(i,j)\in A$, let $\mathcal {W}^k_{ij}$ be the subset of node sets from $\mathcal {W}^k$ for which the induced Steiner cut includes arc (*i*, *j*), i.e.,

$$\begin{aligned} \mathcal {W}^k_{ij}:=\{W \in \mathcal W^k\,{:}\,(i,j)\in \delta ^-(W)\}. \end{aligned}$$

Given a variable vector $\mathbf {v}$ and an index set $\mathcal {I}$, let $v(\mathcal {I})=\sum _{i \in \mathcal {I}} v_i$. Let $\mathbf {c}=(\mathbf {c}^0,\ldots ,\mathbf {c}^k)$ and $\mathbf {T}=(T^1,\dots ,T^k)$. For a given scenario $k \in K$, a node $i\in V{\setminus } T^k$ is referred to as *Steiner node*.

### Related works

The SSTP is NP-hard, as the STP appears as special case for $|K|=1$ and the first-stage cost set to infinity. In the literature, variants and special cases have been addressed by approximation algorithms [15–17, 36].

An algorithm based on fixed-parameter tractability has been introduced in [23], and a genetic algorithm in [19]. The only exact method we are aware of is a two-stage B&C approach based on Benders decomposition, which has been originally proposed in [2]. Very recently, the study of a generalization of the SSTP, namely the stochastic survivable network design problem, along with a more sophisticated implementation, has been given in [27].

In a recent comparison of ILP formulations [41], it is shown that the strongest known formulations for the SSTP are *semi-directed*, i.e., they are defined on *G* in the first and $G_D$ in the second stage. These formulations exploit the property that in an optimal solution $G[E^0_S\cup E^k_S]$ contains a Steiner tree for each $k \in K$, which has a one-to-one correspondence to a Steiner arborescence rooted at *r* on $G_D$. As a consequence, given an (optimal) first-stage solution $E^0_S$, an optimal second-stage solution for each $k\in K$ can be identified by solving a Steiner arborescence problem (SAP) in a modified graph. It is well known that directed formulations based on the STP’s representation as SAP are stronger than their undirected counterparts [11], and the same relation holds between semi-directed and undirected formulations for the SSTP [41]. Unfortunately, as shown in [41] the SSTP cannot be formulated in a purely directed setting.

Consider the following two semi-directed cut formulations, $(\text {SDC}_1)$ and $(\text {SDC}_2)$, studied in [2, 27, 41].
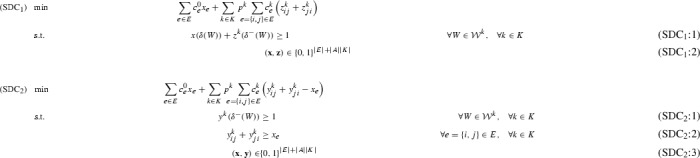


In both formulations, binary variables $x_e$ indicate if edge *e* is chosen as part of the first stage ($x_e=1$) or not ($x_e=0$). A subtle difference exists between the meaning of binary second-stage variables $\mathbf {z}$ in $(\text {SDC}_1)$ and $\mathbf {y}$ in $(\text {SDC}_2)$. In $(\text {SDC}_1)$, for each scenario $k\in K, z^k_{ij}$ indicates if arc (*i*, *j*) is chosen as part of the second stage ($z^k_{ij}=1$) or not ($z^k_{ij}=0$). In $(\text {SDC}_2), y^k_{ij}$ have the same interpretation for $x_e=0, e=\{i,j\}$. Otherwise, they indicate in which direction a first-stage edge can be (potentially) used as part of the Steiner arborescence corresponding to $G[E_S^0\cup E_S^k]$. Linking constraints (SDC$_2$:2) enforce this choice of direction. By optimality, it is guaranteed that per chosen first-stage edge exactly one arc is chosen in each scenario. Note that in an optimal solution a first-stage edge might only be used by a subset of scenarios, but constraints (SDC$_2$:2) imply that for *every* scenario an arc must be chosen (i.e., must be oriented in the second-stage), even though the arc may not be part of the scenario’s Steiner arborescence. In these cases the superfluous cost must be subtracted again in the objective function. Observe that the integrality requirements on $\mathbf {x}$ can be relaxed, as whenever $\mathbf {y}$ is binary, $\mathbf {x}$ will automatically take on a binary value, too. $(\text {SDC}_2)$ provides the advantage that connectivity is modeled by purely directed connectivity cuts (SDC$_2$:1). In the worst case (only first-stage edges are chosen), the presence of undirected variables in (SDC$_1$:1) has the effect that the Linear Programming (LP) relaxation of $(\text {SDC}_1)$ is equivalent to the one of a purely undirected formulation [41].

Despite $(\text {SDC}_2)$ being strictly stronger than $(\text {SDC}_1)$ (this result has been proven in [27], see also [41]), there is a potential shortcoming of that formulation. As already noted, due to the linking constraints (SDC$_2$:2), the arc set induced by $\mathbf {y}^k$ does not form an arborescence in an optimal solution, i.e., the solution induced by $\mathbf {y}^k$ is a union of a Steiner arborescence connecting *r* with $T^k$ and a subset of oriented edges that are purchased in the first-stage. Therefore, the flow-balance inequalities (FB) stating that each Steiner node cannot be a leaf in any optimal solution, 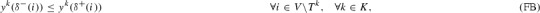 are not valid for $(\text {SDC}_2)$ without further modification. This is unfortunate, as the corresponding flow-balance inequalities for the STP are known to strengthen the LP relaxation of its directed cut formulation (see, e.g, [21, 31]).

In the following, we develop a new ILP formulation that explicitly takes advantage of the flow-balance constraints and whose lower bounds dominate those of all known models from the literature.

## A new ILP formulation

Our new formulation, in which inequalities similar to (FB) hold, is derived on the basis of $(\text {SDC}_1)$. First, copies $\mathbf {x}^k$ of the undirected first-stage variables $\mathbf {x}$ are introduced together with linking constraints $\mathbf {x}=\mathbf {x}^k$ for each scenario $k \in K$. Due to $\mathbf {c}^0\in \mathbb {R}^{|E|}_{\ge 0}$ these equations can be relaxed to inequalities, i.e., $\mathbf {x}\ge \mathbf {x}^k, \forall k \in K$. As discussed during the introduction of $(\text {SDC}_2)$, in an optimal solution a first-stage edge will only be used in at most one direction. We therefore replace undirected edge variables $\mathbf {x}^k$ by corresponding arc variables $\mathbf {w}\in \{0,1\}^{|A||K|}$ and impose constraints $x_e\ge w^k_{ij}+w^k_{ji}$, for all $e=\{i,j\}\in E$, and all $k \in K$, instead.

Each variable $w^k_{ij}$ indicates if the Steiner arborescence of scenario *k* uses the first-stage edge $e=\{i,j\}$ and it also determines its orientation for the given scenario *k*. It follows that in an optimal solution, the arc set induced by variables $\mathbf {z}^k$ and $\mathbf {w}^k$ with values equal to one forms a Steiner arborescence rooted at *r* connecting all terminals from $T^k$. Thus, we obtain a new valid ILP formulation for the SSTP, that we denote by $(\text {SDC}_3)$: 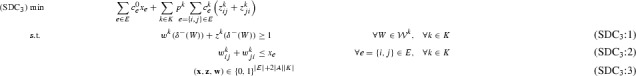


Observe that integrality requirements on $\mathbf {x}$ can be relaxed, as in an optimal solution for any binary $\mathbf {w}$ and $\mathbf {z}, \mathbf {x}$ will automatically take a binary value.

### Comparison between $(\text {SDC}_2)$ and $(\text {SDC}_3)$

In the following, we focus on the strength of the LP relaxation bound of $(\text {SDC}_3)$ and compare it with the corresponding bound for the model $(\text {SDC}_2)$ presented in Sect. 1.1. To this end, we introduce additional notation. Given $\tilde{\mathbf{y}}\in \mathbb {R}^{|A||K|}$, let

$$\begin{aligned} \tilde{\alpha }^k_{ij}:={\left\{ \begin{array}{ll} \frac{\tilde{y}^k_{ij}}{\tilde{y}^k_{ij}+\tilde{y}^k_{ji}} &{}\quad \text {if }\quad \tilde{y}^k_{ij}+\tilde{y}^k_{ji} > 0, \\ 0 &{}\quad \text {otherwise}. \end{array}\right. } \end{aligned}$$

By construction, $\tilde{\alpha }^k_{ij}+\tilde{\alpha }^k_{ji}\in \{0,1\}$, for all $(i,j)\in A$, and all $k \in K$.

#### Definition 2

Let $\varphi$ and $\psi$ denote the following mappings:

$$\begin{aligned}&\varphi : (\tilde{\mathbf{x}},\tilde{\mathbf{y}})\in \mathbb {R}^{|E|+|A||K|} \mapsto (\hat{\mathbf{x}},\hat{\mathbf{w}},\hat{\mathbf{z}}) \in \mathbb {R}^{|E|+2|A||K|}\nonumber \\&\varphi (\tilde{\mathbf{x}},\tilde{\mathbf{y}}):{\left\{ \begin{array}{ll} \hat{x}_e:=\tilde{x}_e &{}\quad \forall e \in E\\ \hat{z}^k_{ij}:=\tilde{y}^k_{ij}-\tilde{\alpha }^k_{ij} \tilde{x}_e &{}\quad \forall (i,j) \in A, e=\{i,j\},\quad \forall k \in K \\ \hat{w}^k_{ij}:=\tilde{\alpha }^k_{ij}\tilde{x}_e &{}\quad \forall (i,j) \in A, e=\{i,j\},\quad \forall k \in K \\ \end{array}\right. } \nonumber \\&\psi : (\hat{\mathbf{x}},\hat{\mathbf{z}},\hat{\mathbf{w}}) \in \mathbb {R}^{|E|+2|A||K|} \mapsto (\tilde{\mathbf{x}},\tilde{\mathbf{y}}) \in \mathbb {R}^{|E|+|A||K|} \nonumber \\&\psi (\hat{\mathbf{x}},\hat{\mathbf{z}},\hat{\mathbf{w}}):{\left\{ \begin{array}{ll} \tilde{x}_e:=\hat{x}_e &{}\quad \forall e \in E\\ \tilde{y}^k_{ij}:=\hat{z}^k_{ij}+\hat{w}^k_{ij}+\frac{1}{2} \left( \hat{x}_{e} - \hat{w}^k_{ij} - \hat{w}^k_{ji}\right) &{}\quad \forall (i,j) \in A, e=\{i,j\},\,\, \forall k \in K \nonumber \\ \end{array}\right. } \end{aligned}$$

The value of a formulation is denoted by $v(\cdot )$, its LP relaxation by prepending “LP-” to its name. Without loss of generality, we assume that any given LP solution is minimal, i.e., no variable can be decreased such that the solution remains feasible and the objective value does not increase. Moreover, as the upper bound constraints of an LP relaxation are redundant in a minimization setting, they are not considered. The following result shows that the two formulations, $(\text {SDC}_2)$ and $(\text {SDC}_3)$, are equally strong.

#### Theorem 1

$$v(\text {LP-SDC}_2)=v(\text {LP-SDC}_3)$$.

#### Proof

$v(\text {LP-SDC}_3)\le v(\text {LP-SDC}_2)$: Let $(\tilde{\mathbf{x}},\tilde{\mathbf{y}})$ be a solution to $(\text {LP-SDC}_2)$. Define $(\hat{\mathbf{x}},\hat{\mathbf{z}},\hat{\mathbf{w}}):=\varphi (\tilde{\mathbf{x}},\tilde{\mathbf{y}})$. Due to the feasibility of $(\tilde{\mathbf{x}},\tilde{\mathbf{y}})$, the constructed point satisfies all bound constraints of $(\text {LP-SDC}_3)$. The choice of $\tilde{\alpha }^k_{ij}$ in particular guarantees that each $\hat{z}^k_{ij}$ is non-negative. Moreover, due to constraints (SDC$_2$:2), $\tilde{y}^k_{ij}+\tilde{y}^k_{ji}=0$ implies $\tilde{x}_e=0$, so $(\tilde{\alpha }^k_{ij}+\tilde{\alpha }^k_{ji}) \tilde{x}_e = \tilde{x}_e$. As a consequence, under mapping $\varphi$ Eqs. (1)–(3) hold for each $(i,j)\in A, e=\{i,j\}$ and $k \in K$:

1 $$\begin{aligned} \hat{z}^k_{ij} + \hat{z}^k_{ji}= & {} \tilde{y}^k_{ij} + \tilde{y}^k_{ji} - \tilde{x}_e \end{aligned}$$

2 $$\begin{aligned} \hat{w}^k_{ij} + \hat{z}^k_{ij}= & {} \tilde{y}^k_{ij} \end{aligned}$$

3 $$\begin{aligned} \hat{w}^k_{ij} + \hat{w}^k_{ji}= & {} \hat{x}_e \end{aligned}$$

Due to (1), the objective values associated to $(\tilde{\mathbf{x}},\tilde{\mathbf{y}})$ by $(\text {LP-SDC}_2)$ and to $(\hat{\mathbf{x}},\hat{\mathbf{z}},\hat{\mathbf{w}})$ by $(\text {LP-SDC}_3)$ are equal. Due to Eqs. (2) and (3), $(\hat{\mathbf{x}},\hat{\mathbf{z}},\hat{\mathbf{w}})$ satisfies both (SDC$_3$:1) and (SDC_3_:2), and is thus feasible for $(\text {LP-SDC}_3)$.

$v(\text {LP-SDC}_2)\le v(\text {LP-SDC}_3)$: Let $(\hat{\mathbf{x}},\hat{\mathbf{z}},\hat{\mathbf{w}})$ be a solution to $(\text {LP-SDC}_3)$. Define $(\tilde{\mathbf{x}},\tilde{\mathbf{y}}):=\psi (\hat{\mathbf{x}},\hat{\mathbf{z}},\hat{\mathbf{w}})$. Due to the feasibility of $(\hat{\mathbf{x}},\hat{\mathbf{z}},\hat{\mathbf{w}})$, the constructed point satisfies all bound constraints of $(\text {LP-SDC}_2)$. Under mapping $\psi$, Eq. (1) holds, and thus also in this case the objective values of both points are equal under their respective objective function. Moreover, due to $z^k_{ij}+z^k_{ji}\ge 0$ and (1), (SDC$_2$:2) are satisfied. Finally, $(\tilde{\mathbf{x}},\tilde{\mathbf{y}})$ satisfies (SDC$_2$:1) since $(\hat{\mathbf{x}},\hat{\mathbf{z}},\hat{\mathbf{w}})$ satisfies (SDC$_3$:1) and (SDC_3_:2):
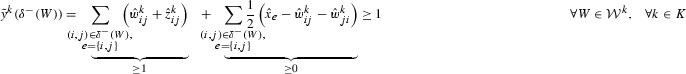


Thus $(\tilde{\mathbf{x}},\tilde{\mathbf{y}})$ is feasible for $(\text {LP-SDC}_2)$. $\square$

The latter result may appear discouraging, since it shows that the two basic models, $(\text {SDC}_2)$ and $(\text {SDC}_3)$, provide the same quality of LP relaxation bounds, whereas the second one comes at the cost of introducing additional |*A*||*K*| variables $\mathbf {w}$. However, the following section demonstrates that the model $(\text {SDC}_3)$ has significant advantages over $(\text {SDC}_2)$, due to the modeling of strong flow-balance constraints. Moreover, in Sects. 3.1–3.3, we show how to construct alternative methods for computing lower bounds and for applying variable fixing. These methods specifically exploit the property that for each scenario $k\in K$ the corresponding second-stage solution forms a Steiner arborescence in $(\text {SDC}_3)$.

### Flow-balance constraints

Formulations $(\text {SDC}_2)$ and $(\text {SDC}_3)$ can be strengthened by adding variants of the STP’s flow-balance inequalities, (SDC$_2$:FB) and (SDC$_3$:FB), respectively. 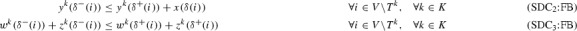


Constraints (SDC$_2$:FB) ensure that the in-degree of a Steiner node in the second-stage is not greater than its out-degree, unless the node is adjacent to an edge that has been purchased in the first-stage. A stronger version of these constraints can be imposed for model $(\text {SDC}_3)$ due to the fact that the second-stage solution is now modeled as a Steiner arborescence. Constraints (SDC$_3$:FB) ensure that after orienting the solution in the second-stage, each node $i \not \in T^k$, cannot be a leaf in the second-stage, if scenario *k* occurs.

**Fig. 1:**
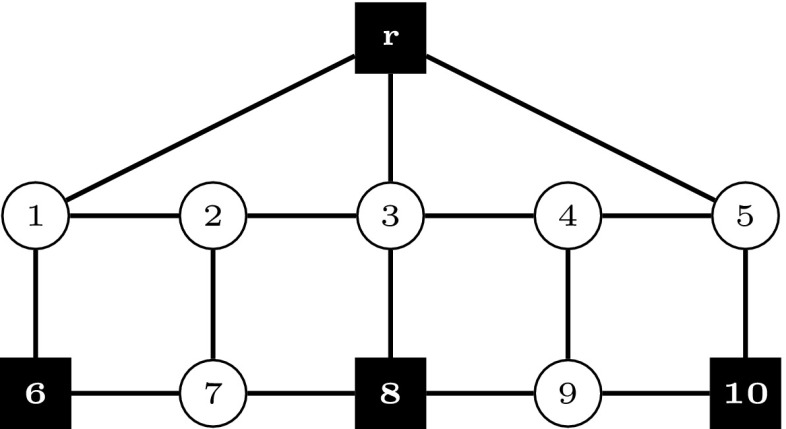
Example. $K=\{1,2\}, p^1=p^2=0.5, T^1=\{r,6,8\}, T^2=\{r,8,10\}$, for each $e\in E, c^0_e=2$ if incident to a terminal, else $c^0_e=1$. Second stage costs are set to $\mathbf {c}^1=1.4 \mathbf {c}^0$ and $\mathbf {c}^2=1.1 \mathbf {c}^0$, respectively

In the following, we compare the strength of models $(\text {SDC}_2)$ and $(\text {SDC}_3)$ with flow-balance constraints. Theorems 2–3 show that adding flow-balance constraints in the respective models can lead towards a strictly stronger formulation, whereas Theorem 4 proves that the strongest LP relaxation bounds are obtained by model $(\text {SDC}_3^{\text {FB}})$. All theorems make use of the example shown in Fig. 1. In this instance, we are given two scenarios ($K=\{1,2\}$), with second-stage costs computed from first-stage costs based on a fixed inflation factor. Terminals are represented by black squares. In Fig. 1, the union of terminals over all scenarios is shown, i.e., $\bigcup _{k\in K}T^k$.

**Fig. 2:**
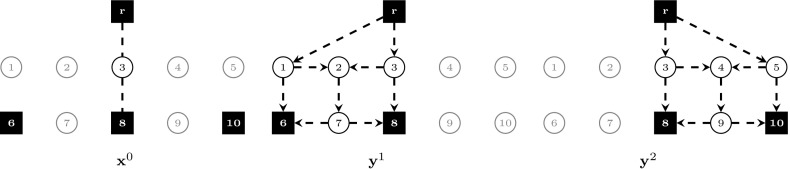
Fractional optimal LP solution to $(\text {SDC}_2)$ for the example shown in Fig. 1, $v(\text {LP-SDC}_2)=8.875$. The solution is drawn separately according to stage and scenario. Arcs/edges are omitted for LP values equal to 0, drawn dashed for 0.5, and drawn solid for 1

**Fig. 3:**
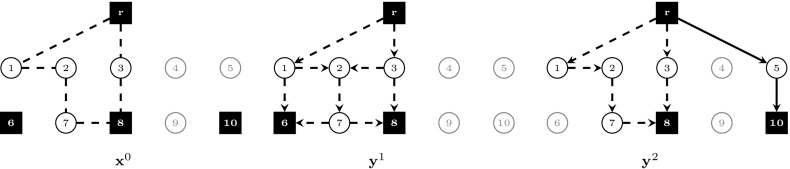
Fractional optimal LP solution to $(\text {SDC}_2^{\text {FB}})$ for the example shown in Fig. 1, $v(\text {LP-SDC}_2^{\text {FB}})=8.95$. The solution is drawn separately according to stage and scenario. Arcs/edges are omitted for LP values equal to 0, drawn dashed for 0.5, and drawn solid for 1

**Fig. 4:**
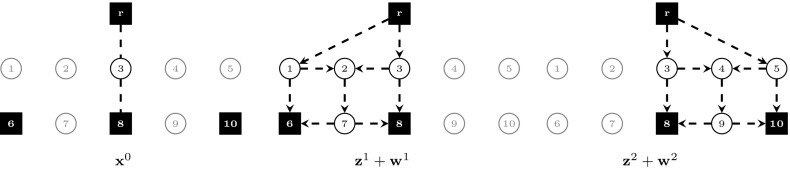
Fractional optimal LP solution to $(\text {SDC}_3)$ for the example shown in Fig. 1, $v(\text {LP-SDC}_3)=8.875$. The solution is drawn separately according to stage and scenario. Arcs/edges are omitted for LP values equal to 0, drawn dashed for 0.5, and drawn solid for 1

Figures 2, 3 and 4 show optimal LP solutions to the instance from Fig. 1 for different formulations. Each solution is displayed in three separate subfigures, one for the first stage and two for the second stage, one for each scenario. An edge/arc is omitted if the LP value of the associated variable is 0. Otherwise, if the LP value is 0.5 or 1, it is drawn dashed or solid, respectively. When displaying LP solutions for $(\text {SDC}_3)$ and $(\text {SDC}_3^{\text {FB}})$, note that for all $k \in K, (i,j)\in A$, either $z^k_{ij}=0$ or $w^k_{ij}=0$. Moreover, for all $e=\{i,j\}\in E, x_e>0$ implies $z^k_{ij}=0$ for all $k \in K$. Thus for these formulations only one graph for each scenario is shown, based on the LP values of $\mathbf {z}^k+\mathbf {w}^k$.

#### Theorem 2

$v(\text {LP-SDC}_2)\le v(\text {LP-SDC}_2^{\text {FB}})$ and there exist instances in which the inequality is strict.

#### Proof

Figures 2 and 3 show optimal solutions to $(\text {LP-SDC}_2)$ and $(\text {LP-SDC}_2^{\text {FB}})$, respectively. The former solution violates (SDC$_2$:FB) for $k=1$, node $i=2$ and $k=2$, node $i=4$, while all inequalities of this type are satisfied for the latter. Moreover, $v(\text {LP-SDC}_2)=8.875<8.95=v(\text {LP-SDC}_2^{\text {FB}})$ in the example. $\square$

#### Theorem 3

$v(\text {LP-SDC}_3)\le v(\text {LP-SDC}_3^{\text {FB}})$ and there exist instances in which the inequality is strict.

#### Proof

**Fig. 5:**
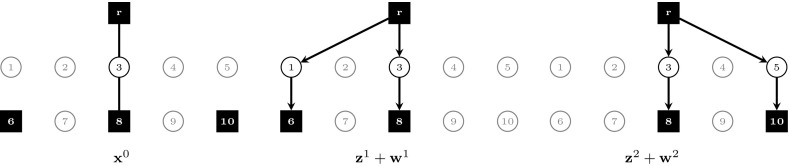
Integral optimal LP solution to $(\text {SDC}_3^{\text {FB}})$ for the example shown in Fig. 1, $v(\text {LP-SDC}_3^{\text {FB}})=9$. The solution is drawn separately according to stage and scenario. Arcs/edges are omitted for LP values equal to 0 and are drawn solid for 1

Figures 4 and 5 show optimal solutions to $(\text {LP-SDC}_3)$ and $(\text {LP-SDC}_3^{\text {FB}})$, respectively. The former solution violates (SDC$_3$:FB) for $k=1$, node $i=2$ and $k=2$, node $i=4$, while all inequalities of this type are satisfied for the latter. Moreover, $v(\text {LP-SDC}_3)=8.875<9=v(\text {LP-SDC}_3^{\text {FB}})$ in the example. $\square$

#### Theorem 4

$v(\text {LP-SDC}_2^{\text {FB}})\le v(\text {LP-SDC}_3^{\text {FB}})$ and there exist instances in which the inequality is strict.

#### Proof

Let $(\hat{\mathbf{x}},\hat{\mathbf{z}},\hat{\mathbf{w}})$ be a solution to $(\text {LP-SDC}_3^{\text {FB}})$. Define $(\tilde{\mathbf{x}},\tilde{\mathbf{y}}):=\psi (\hat{\mathbf{x}},\hat{\mathbf{z}},\hat{\mathbf{w}})$. By Theorem 1, $(\tilde{\mathbf{x}},\tilde{\mathbf{y}})$ is feasible for $(\text {LP-SDC}_2)$. It remains to show that the point satisfies (SDC$_2$:FB). As indicated by (4 5)–(6), this is the case under mapping $\psi$ if $(\hat{\mathbf{x}},\hat{\mathbf{z}},\hat{\mathbf{w}})$ satisfies (SDC$_3$:FB). Thus $(\tilde{\mathbf{x}},\tilde{\mathbf{y}})$ is feasible for $(\text {LP-SDC}_2^{\text {FB}})$.

4 $$\begin{aligned}&\hat{w}^k(\delta ^-(i))+\hat{z}^k(\delta ^-(i)) \le \hat{w}^k(\delta ^+(i))+\hat{z}^k(\delta ^+(i))\end{aligned}$$

5 $$\begin{aligned}&\iff \hat{w}^k(\delta ^-(i))+\hat{z}^k(\delta ^-(i)) + \frac{1}{2}(\hat{x}_e - \hat{w}_{ij} - \hat{w}_{ji})\le \hat{w}^k(\delta ^+(i))+\hat{z}^k(\delta ^+(i))\nonumber \\&\qquad \qquad \qquad \qquad \qquad \qquad +\, \frac{1}{2}(\hat{x}_e - \hat{w}_{ij} - \hat{w}_{ji}) \end{aligned}$$

6 $$\begin{aligned}&\iff \tilde{y}^k(\delta ^-(i)) \le \tilde{y}^k(\delta ^+(i)) \end{aligned}$$

In order to show that there exist instances in which the inequality is strict, Figs. 3 and 5 depict the optimal solutions to $(\text {LP-SDC}_2^{\text {FB}})$ and $(\text {LP-SDC}_3^{\text {FB}})$, respectively. One can verify that in each case, all respective flow-balance inequalities are satisfied. Moreover, $v(\text {LP-SDC}_2^{\text {FB}})= 8.95< 9=v(\text {LP-SDC}_3^{\text {FB}})$. $\square$

In summary, one can observe that in case no first-stage edges are chosen, (SDC$_2$:FB) have the same effect as in the SAP. This behavior can be compared to $(\text {SDC}_1)$, which may be as strong as $(\text {SDC}_2)$ in the same scenario. In contrast, (SDC$_3$:FB) can potentially improve the LP bound even if all edges are chosen in the first-stage.

### Hierarchy of formulations

**Fig. 6:**
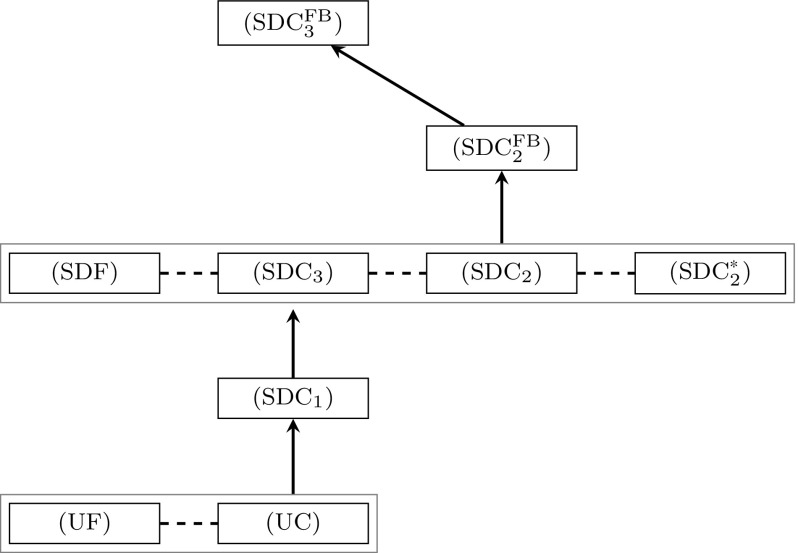
Hierarchy of formulations for the SSTP. Surrounding boxes and dashed lines indicate equivalence, while directed arcs indicate that the target formulation is stronger than the source formulation

The theoretical results of this section are summarized in Fig. 6, in which we augment the recent findings from [41] with our new results. For the sake of brevity, some of the formulations from this hierarchy are not shown in this article. These are: $(\text {UF})$ and $(\text {UC})$ that denote the undirected flow and cut formulations, and $(\text {SDF})$ which denotes the semi-directed flow-formulation. $(\text {SDC}^*_2)$ denotes a variant of $(\text {SDC}_2)$ with aggregated coefficients in the objective function. The models have been studied in [41], where the lower three levels of the shown hierarchy (except for the model $(\text {SDC}_3)$) have been proven. In this article we introduce the formulation $(\text {SDC}_3)$ and show that it is equally strong as the other strongest models from [41]. Our new models $(\text {SDC}_2^{\text {FB}})$ and $(\text {SDC}_3^{\text {FB}})$ are the formulations $(\text {SDC}_2)$ and $(\text {SDC}_3)$ augmented with flow-balance inequalities (SDC$_2$:FB) and (SDC$_3$:FB), respectively. We show that $(\text {SDC}_2^{\text {FB}})$ and $(\text {SDC}_3^{\text {FB}})$ further improve the LP relaxations bounds, with the strongest ones being obtained with the $(\text {SDC}_3^{\text {FB}})$ model.

## Algorithmic framework

Given the results of Sect. 2, we decided to build our algorithmic framework based on formulation $(\text {SDC}_3^{\text {FB}})$. It is well-known that the size of two-stage stochastic optimization models becomes prohibitive for a large number of scenarios. Hence, in order to develop a computationally competitive approach, naturally one has to rely on a decomposition framework.

Two types of decomposition techniques are commonly employed for this task: Benders decomposition (see, e.g., [1, 5]) and Lagrangian relaxation (see, e.g., [9, 39]). These two approaches can be seen as dual to each other [32, 33], as the former decomposes the problem by stage, while the latter decomposes by scenario. Naturally, both of them can be used as stand-alone procedures for solving a large-scale stochastic optimization problem. However, using both Lagrangian and Benders decomposition in a combined framework opens up further possibilities to obtain the benefits of both procedures, as the problem at hand can be attacked from multiple angles, in order to exploit different types of problem-structures [38].

**Fig. 7:**
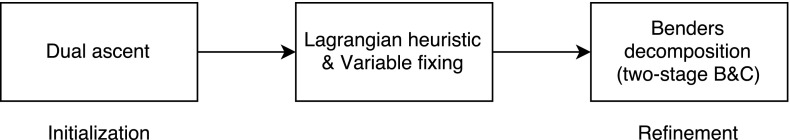
Algorithmic framework

Thus our framework combines these two decomposition approaches. Furthermore, due to similarities between the SSTP and its deterministic variant, a third option for computing lower bounds appears promising: a dual ascent procedure, which constructs an initial dual solution in a greedy scheme (see [28, 30, 40]). For the STP and its variants, such procedures are known empirically to obtain high quality bounds, which in some cases are even tight enough to solve the corresponding problem to optimality in a branch-and-bound (B&B) procedure [26, 28, 31]. In the framework proposed in this article, we chose the configuration of these three techniques as shown in Fig. 7.

The central component of our approach is a Lagrangian-based heuristic that computes valid lower and upper bounds and performs variable fixing based on the dual information. This Lagrangian procedure is warm-started by a dual ascent heuristic (specifically derived for the SSTP). Finally, an additional refinement of the computed lower bound is performed by a Benders decomposition framework (actually, a two-stage B&C similar to the one proposed in [2, 27]).

Note that the chosen sequence of execution is a natural one, as the methods are arranged based on the computational complexity (see Sects. 3.1–3.3 for details). The communication between each distinct phase is performed by passing on primal and dual solutions. Most importantly, the dual solution computed by the dual ascent procedure is used as an initial set of Lagrange dual multipliers to warm-start a subgradient algorithm. Similarly, a subset of all computed Lagrangian dual solutions is used to generate valid cutting planes, which are then used to initialize the two-stage B&C procedure.

Moreover, although $(\text {SDC}_3^{\text {FB}})$ offers tighter bounds than $(\text {SDC}_3)$, we found it beneficial to only make use of the flow-balance constraints in the final refinement phase. The main reason is that in our implementation, up to this point we focus mainly on the use of fast dual heuristics, in which the inclusion of such constraints is a non-trivial aspect. Even in state-of-the-art algorithmics frameworks for the STP, their inclusion is usually avoided [28, 31] for simplicity reasons. Furthermore, the tighter bounds provided by $(\text {SDC}_3^{\text {FB}})$ are mainly useful when attempting to solve the instance to optimality, i.e., in the refinement phase.

In the remainder of this section we provide the algorithmic and implementational details of this new method.

### Lagrangian relaxation and reduced cost fixing

The Lagrangian relaxation of model $(\text {SDC}_3)$ is obtained by relaxing constraints (SDC_3_:2) and adding them to the objective function as penalty terms. The associated set of non-negative *Lagrangian dual multipliers* is denoted by $\varvec{\lambda }$. For each value of $\varvec{\lambda }$, the resulting *Lagrangian relaxation* yields a valid lower bound for $(\text {SDC}_3)$, and is given as follows. 
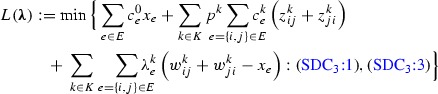


After rearranging the terms in the objective function and moreover defining the *Lagrangian cost* as $\tilde{c}_e:=c^0_e-\sum _{k \in K} \lambda ^k_e, e\in E$, we obtain the following, simplified representation.
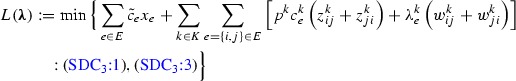


The problem decomposes into $|K|+1$ independent subproblems, one in $\mathbf {x}$ and the others in $\mathbf {z}^k,\mathbf {w}^k$ for $k \in K$. 
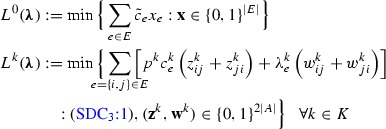


The *Lagrangian dual problem*, which corresponds to finding the best lower bound, is then stated as:

$$\begin{aligned} (\text {SDC}^{LD}_3)\quad&\max _{\varvec{\lambda \ge 0}} \Big \{L^0(\varvec{\lambda })+\sum _{k\in K}L^k(\varvec{\lambda })\Big \}. \end{aligned}$$

It is easy to see that $L^0(\varvec{\lambda })$ can be computed by inspection. For $L^k(\varvec{\lambda })$, there exists an optimal solution in which either $z^k_{ij}$ or $w^k_{ij}$ (or none of them) will be chosen for each $k \in K$—and this choice depends solely on the property if $p^k c^k_{e} < \lambda ^k_e$ holds or not. Thus the computation of $L^k(\varvec{\lambda })$ is equivalent to solving an instance of the SAP, i.e., given terminals $T^k$ and arc costs $\min \{p^k c^k_{e}, \lambda ^k_e\}$ for all $(i,j) \in A, e=\{i,j\}$, the objective is to find a minimum-cost arborescence rooted at *r* which contains a directed path from *r* to each terminal $t \in T^k$. Furthermore, in a minimal optimal solution to $L^k(\varvec{\lambda })$, flow-balance inequalities will be satisfied.

The following result provides a further justification for choosing the model $(\text {SDC}_3)$ in our approach, as the Lagrangian dual bounds obtained from $(\text {SDC}_3)$ can be even stronger than the LP relaxation bounds from $(\text {SDC}_3^{\text {FB}})$.

#### Theorem 5

$$v(\text {LP-SDC}_3^{\text {FB}})\le v(\text {SDC}^{LD}_3)= v(\text {SDC}_3)$$.

#### Proof

The LP relaxation of $L^k(\varvec{\lambda })$ augmented with flow-balance inequalities (SDC$_3$:FB) does not have the *integrality property*, and therefore the optimal solution to $(\text {SDC}^{LD}_3)$ may yield a stronger bound. Moreover, the Lagrangian dual bound of $(\text {SDC}_3)$ is equal to the optimal solution value, which follows from the fact that no integrality conditions are imposed on variables $\mathbf {x}$ that only appear in the relaxed linking constraints (SDC_3_:2) (see Theorem 8.2 from [4] for further details). $\square$

#### Variable fixing based on reduced costs

Variable fixing based on reduced costs is an indispensable tool in many modern primal–dual solution frameworks [26, 31]. In this section we address conditions under which first- and second-stage variables of $(\text {SDC}_3)$ can be eliminated, given valid Lagrangian dual multipliers $\bar{\varvec{\lambda }}$.

In addition to the Lagrangian cost $\tilde{\mathbf{c}}^0$ associated to the first-stage variables $\mathbf {x}$, for each $k \in K$ we consider the LP relaxation of the Lagrangian subproblem for computing $L^k(\bar{\varvec{\lambda }})$ in order to obtain reduced cost also for the second-stage variables $\mathbf {z}^k$ and $\mathbf {w}^k$. The associated LP dual is given as follows. We use $\underline{L}^k(\bar{\varvec{\lambda }})$ to denote the corresponding lower bound.

$$\begin{aligned} \underline{L}^k(\bar{\varvec{\lambda }}):=\;&\text {max}\;&\sum _{{W \in \mathcal W^k}} \beta ^k_W&\\&\text {s.t.}&\beta \left( \mathcal {W}^k_{ij}\right)&\le \min \{p^k c^k_{e},\bar{\lambda }^k_{e}\}&\forall (i,j) \in A, e=\{i,j\}\\& & \varvec{\beta }^k \in \mathbb {R}_{\ge 0}^{|\mathcal {W}^k|}&\end{aligned}$$

Variables $\varvec{\beta }^k$ are non-negative dual multipliers associated to each connectivity cut constraint  (SDC$_3$:1) Given a feasible solution $\bar{\varvec{\beta }}^k$, the reduced cost of the packing constraints in the dual are

$$\begin{aligned} \tilde{c}^k_{ij}:=\min \left\{ p^k c^k_{e}, \bar{\lambda }^k_e\right\} - \bar{\beta }\left( \mathcal {W}^k_{ij}\right)&\forall (i,j)\in A, e=\{i,j\}. \end{aligned}$$

Let the vector of Lagrangian cost and LP reduced cost be $\tilde{\mathbf{c}}:=(\tilde{\mathbf{c}}^0,\dots ,\tilde{\mathbf{c}}^k)$. The value of $L^0({\bar{\varvec{\lambda }}})$ together with a lower bound $\underline{L}^k(\bar{\varvec{\lambda }})$ to $L^k(\bar{\varvec{\lambda }})$ for each $k\in K$ yields a valid lower bound to the original problem, i.e., $LB:=\underline{L}(\bar{\varvec{\lambda }})=L^0(\bar{\varvec{\lambda }})+\sum _{k\in K} \underline{L}^k(\bar{\varvec{\lambda }})$. Based on Lagrangian duality (see, e.g., [39]), this information in combination with a valid upper bound $UB$ allows variables to be fixed either to one or zero. For example, if $LB +\tilde{c}^k_{ij}> UB$, both $z^k_{ij}$ and $w^k_{ij}$ can be fixed to zero, as the cost of an optimal solution in which either of these two variables are set to one will exceed $UB$. Conversely, observe that fixing variables to one is only possible for first-stage variables $x_e$ for each $e \in E,$ as second-stage reduced cost are non-negative. However, as will be shown, fixing variables to zero is usually a more promising strategy anyway, as in this case various structural properties of an optimal solution can be exploited.

We begin with a simple observation based on the packing constraints in the stated dual. If a second-stage variable $z^k_{ij}$ or $w^k_{ij}$ is set to one for an arc $(i,j)\in A, e=\{i,j\}$, depending if either $z^k_{ij}$ or $w^k_{ij}$ is fixed, additional reduced cost are incurred due to the $\min$-expression. If $z^k_{ij}$ is fixed to one, then in addition to $\tilde{c}^k_{ij}, \max \{0,p^k c^k_{e}-\bar{\lambda }^k_e\}$ is paid. Conversely, if $w^k_{ij}$ is fixed to one, then $\max \{0,\bar{\lambda }^k_e-p^k c^k_{e}\}$ is paid in addition.

Additional incurred reduced cost can be inferred based on well-known arguments for the SAP. By minimality, each arc $(i,j)\in A$ part of an optimal arborescence corresponding to $L^k(\bar{\varvec{\lambda }})$ must lie on a directed path from *r* to some terminal $t \in T^k$. Based on this property, it can be shown that setting an arc variable to one will incur the reduced cost of all arcs on this path [6, 31]. As the optimal arborescence is not known, a frequently applied approach is to obtain an efficiently computable underestimation of these costs [6, 10, 26, 31]. Let $\tilde{d}^k_{ij}$ denote the cost of a shortest path from *i* to *j* on $G_D$ computed based on $\tilde{\mathbf{c}}^k$. The incurred reduced cost can be bounded from below by computing the shortest path based on reduced cost from *r* to *i* and from *j* to the closest terminal $t\in T^k$, i.e.,

$$\begin{aligned} \tilde{D}^k_{ij}:=\tilde{d}^k_{ri}+\tilde{c}^k_{ij} + \min _{t \in T^k} \tilde{d}^k_{jt} \quad \forall k \in K. \end{aligned}$$

Note that similar reasoning applies if a first-stage edge variable $x_e, e \in E$ is set to one, as at least one second-stage arc corresponding to this first-stage edge must be part of an optimal solution. However, the direction of this arc is usually not known (either $w^\ell _{ij}=1$ or $w^\ell _{ji}=1$ for some $\ell \in K$). In this case the cheaper direction is a valid underestimation of the reduced cost of this path.

$$\begin{aligned} \tilde{D}^k_e:={\min \left\{ \tilde{D}^k_{ij},\tilde{D}^k_{ji}\right\} +\max \left\{ 0,\bar{\lambda }^k_e-p^k c^k_{e}\right\} }, \quad \forall k \in K, e=\{i,j\}. \end{aligned}$$

Based on these definitions and observations, Proposition 1 state conditions when variables $\mathbf {x}, \mathbf {w}$, and $\mathbf {z}$ can be fixed to zero.

##### Proposition 1

Given a lower bound LB, associated reduced cost $\varvec{\tilde{c}}$ and dual multipliers $\bar{\varvec{\lambda }}$, as well as an upper bound $UB$, a variable $z^k_{ij}$ can be fixed to zero if

7 $$\begin{aligned} LB + \tilde{D}^k_{ij} + \max \left\{ 0,p^k c^k_{e}-\bar{\lambda }^k_e\right\} > \text {{ UB}} \end{aligned}$$

holds. Similarly, variable $w^k_{ij}$ can be fixed to zero if

8 $$\begin{aligned} \text {{ LB}}+ \tilde{D}^k_{ij} + \max \left\{ 0,\bar{\lambda }^k_e-p^k c^k_{e}\right\} + \max \left\{ 0,\tilde{c}^0_{e}\right\} > \text {{ UB}} \end{aligned}$$

holds. Finally, variable $x_e$ can be fixed to zero if

9 $$\begin{aligned} \text {{ LB}}+ \min _{k \in K} \tilde{D}^k_{e} + \max \left\{ 0,\tilde{c}^0_{e}\right\} > \text {{ UB}} \end{aligned}$$

holds.

##### Proof

The correctness of the given statements follows from the discussions in the previous paragraphs. Moreover, conditions (8) and (9) exploit the fact that if $\tilde{c}^0_e \le 0$, then the corresponding first-stage edge *e* will already be chosen in an optimal solution of $L^0(\bar{\varvec{\lambda }})$, and thus its reduced cost can be set to zero. $\square$

Conditions (8) and (9) consider only one scenario at a time. Consequently, they can be strengthened based on the property that in an optimal solution, in almost all cases, a first-stage edge needs to to be used in multiple scenarios in order to pay off. More formally, if $e\in E$ is part of an optimal solution’s first stage, then there exists a set of scenarios $K^* \subseteq K$ such that $\sum _{k\in K^*} p^k c^k_e \ge c^0_e$. For each $k \in K^*$ the same path-based arguments apply as made for Proposition 1. The following two conditions again employ an underestimation of the incurred reduced cost based on the solution of knapsack problems in minimization form.

##### Proposition 2

Given a lower bound LB, associated reduced cost $\varvec{\tilde{c}}$ and dual multipliers $\bar{\varvec{\lambda }}$, as well as an upper bound $UB$, a variable $x_e$ can be fixed to zero if

10 $$\begin{aligned} \text {{ LB}}+ \min _{K^* \subseteq K} \Big \{\sum _{k \in K^*} \tilde{D}^k_{e} : \sum _{k\in K^*} p^k c^k_e \ge c^0_e\Big \} + \max \left\{ 0,\tilde{c}^0_e\right\} > \text {{ UB}} \end{aligned}$$

holds. Similarly, variable $w^k_{ij}$ can be fixed to zero if

11 $$\begin{aligned}&\text {{ LB}}+ \tilde{D}^{k}_{ij} + \max \left\{ 0,\bar{\lambda }^k_e-p^k c^k_{e}\right\} \nonumber \\&\quad +\, \min _{K^* \subseteq K{\setminus }\{k\}} \Big \{\sum _{\ell \in K^*} \tilde{D}^\ell _{e} : p^k c^k_{e} + \sum _{\ell \in K^*} p^\ell c^\ell _e\ge c^0_e\Big \}+\max \left\{ 0,\tilde{c}^0_e\right\} > \text {{ UB}}\qquad \end{aligned}$$

##### Proof

For condition (10), let $K'\subseteq K$ be the optimal set of scenarios that uses edge *e* in the first-stage, given that $x_e=1$. From the path-based discussions in the previous paragraphs, $\sum _{k\in K'} \tilde{D}^k_e$ is a valid underestimation of the incurred reduced cost over all scenarios. By definition, it holds that $\sum _{k\in K'} p^k c^k_e \ge c^0_e$ and $\sum _{k\in K^*} p^k c^k_e \ge c^0_e$. Therefore $\sum _{k\in K^*} \tilde{D}^k_e\le \sum _{k\in K'} \tilde{D}^k_e$, and consequently $\sum _{k\in K^*} \tilde{D}^k_e$ is also an underestimation. The argument for condition (11) is equivalent, except that scenario $\ell \in K$, is forced into the knapsack, and the direction in which *e* is used in scenario $\ell$ is fixed.    $\square$

In order to compute all required shortest paths, for each scenario we need two executions of Dijkstra’s algorithm using reduced cost $\tilde{\mathbf{c}}$ as arc lengths: One execution is on $G_D$ using *r* as source, and the other one is on a modified version of $G_D$, in which all arcs are inverted and an artificial source node has been added; this node is connected to each terminal $T^k{\setminus }\{r\}$. This requires $\mathcal {O}(|K|(|A| + |V| \log |V|))$. In order to apply conditions (10) and (11), integrality is relaxed and the so-called Dantzig bound is used. The resulting LP can be solved in $O(|K|\log |K|)$ by choosing elements in ascending order based on their utility ratio $\tilde{D}^k_{e}/(p^k c^k_{e})$ for all $k \in K$. Both for the computation of shortest paths and knapsacks, existing variable fixing is taken into account.

### Warmstart using dual ascent

In order to accelerate the convergence of a Lagrangian-based decomposition approach, in some cases it is essential to initialize the procedure with a suitable choice of Lagrangian dual multipliers (see, e.g., [12, 13] for details on the relationship between dual ascent methods and Lagrangian relaxation). To this end, we propose to run an alternative dual-based approach, namely, a dual ascent procedure. The major benefits of this procedure are: (i) the obtained dual solution $\bar{\varvec{\lambda }}$ is a feasible starting point for $(\text {SDC}^{LD}_3)$, (ii) the lower bound computed in each step increases monotonically, thus providing fast convergence, and (iii) it can be performed efficiently (the algorithm runs in $\mathcal {O}(\sum _{k \in K}|A|\min \{|A|,|T^k||V|\})$ time).

The STP admits the heuristic computation of LP relaxation bounds via the dual ascent procedure by Wong [40]. Although this type of method does not provide tight guarantees on the quality of the computed lower bound, empirically, the bound is usually quite close to the optimum.

Using an appropriate model as a starting point, this approach can be extended seamlessly to the SSTP. Let $(\text {SDC}^D_3)$ denote the dual of $(\text {SDC}_3)$ after relaxing the integrality constraints (SDC$_3$:3). Let $\varvec{\beta }$ and $\varvec{\lambda }$ be the dual multipliers associated to  (SDC$_3$:1) and (SDC_3_:2), respectively.
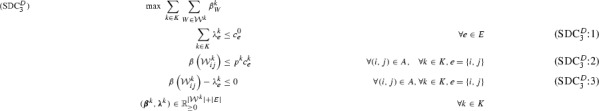


The dual can be simplified into a *condensed dual* $(\text {SDC}^{D'}_3)$, in which variables $\varvec{\lambda }$ are eliminated by aggregating inequalities (SDC$^D_3$:3) and combination with (SDC$^D_3$:1). For any solution $\bar{\varvec{\beta }}\in (\text {SDC}^{D'}_3)$, a solution $(\bar{\varvec{\beta }},\bar{\varvec{\lambda }})\in (\text {SDC}^D_3)$ of equal objective value can be constructed by setting $\bar{\lambda }^k_e=\max \{\bar{\beta }(\mathcal {W}^k_{ij}),\bar{\beta }(\mathcal {W}^k_{ji})\}$ for each $e=\{i,j\}\in E$ and $k \in K$.
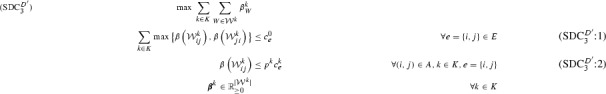


Algorithm 1 lists the pseudocode of our dual ascent procedure that computes a heuristic solution to $(\text {SDC}^{D'}_3)$ and extends it to a feasible solution to $(\text {SDC}^D_3)$. Starting from the initial solution $\bar{\varvec{\beta }}=\mathbf {0}$, in each iteration one dual variable $\beta ^k_W=0$ is increased to its maximum possible value while preserving dual feasibility. As dual variables $\varvec{\beta }$ are exponential in number, they are only tracked implicitly by the algorithm according to the reduced cost of constraints (SDC$^{D'}_3$:1) and (SDC$^{D'}_3$:2), $\tilde{c}^0_{e}:=c^0_{e} - \sum _{k \in K}\max \{\beta (\mathcal {W}^k_{ij}),\beta (\mathcal {W}^k_{ji})\}$ and $\tilde{c}^k_{ij}:=p^k c^k_{ij} - \beta (\mathcal {W}^k_{ij})$, respectively. Similarly, the objective value of the constructed dual solution is tracked as variable $LB$.
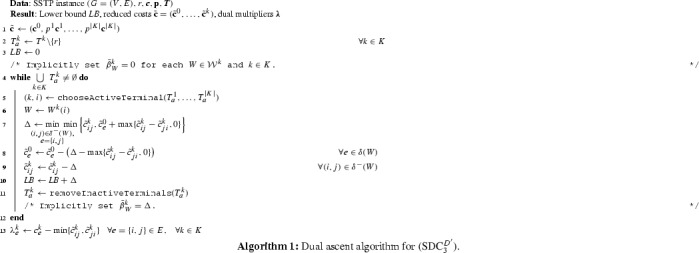


The selection of $\beta ^k_W$ is performed as follows. First, recall that *W* induces a Steiner cut w.r.t. *k*, i.e., $r\notin W$ and $T^k\cap W\ne \emptyset$. For an increase of $\beta ^k_W$ to be feasible, none of the constraints (SDC$^{D'}_3$:2) associated to its directed cutset $\delta ^-(W)$ can be tight. Thus, if only constraints (SDC$^{D'}_3$:2) would be considered, the maximum feasible increase of $\beta ^k_W$ corresponds to the minimum reduced costs of the cutset’s arcs.

Concerning constraints (SDC$^{D'}_3$:1), the same does not hold for the undirected cutset $\delta (W)$ due to the max-expression therein. Consider the case where $\tilde{c}^0_e=0$ for some $e=\{i,j\}\in \delta (W)$. The associated constraint does not prevent a further increase of $\beta ^k_W$ if for the arc $(i,j)\in \delta ^-(W)$ associated to the edge, $\beta (\mathcal {W}^k_{ij})<\beta (\mathcal {W}^k_{ji})$ holds. In this case, in addition to an increase by $\tilde{c}^0_e, \beta ^k_W$ can further be increased by $\beta (\mathcal {W}^k_{ji})-\beta (\mathcal {W}^k_{ij})$, or equivalently, by $\tilde{c}^k_{ij}-\tilde{c}^k_{ji}$.

The stated properties can be represented in terms of subgraphs. Let the *saturation graph* per scenario $k \in K$ be defined as

$$\begin{aligned} G^k_S:= & {} G_D\left[ A^k_S\right] \text { for } A^k_S=\left\{ (i,j) \in A : \tilde{c}^k_{ij}=0 \vee \tilde{c}^0_{e}\right. \\&\left. \quad +\,\max \left\{ \tilde{c}^k_{ij}-\tilde{c}^k_{ji},0\right\} =0, {e=\{i,j\}}\right\} . \end{aligned}$$

The arcs in $A^k_S$ are referred to as saturated w.r.t. *k*. Furthermore, let

$$\begin{aligned} W^k(i):=\left\{ j \in V : \text{ there } \text{ exists } \text{ a } \text{ directed } \text{ path } \text{ from } \text{ j } \text{ to } \text{ i } \text{ in } G^k_S\right\} . \end{aligned}$$

The algorithm maintains a set of active terminals $T^k_a\subseteq T^k{\setminus }\{r\}$ for each $k \in K$. A terminal *i* is said to be *active* for *k* if $r \notin W^k(i)$ and $|W^k(i)\cap T^k_a|=1$. The former condition implies that $W^k(i)$ induces a Steiner cut, while the latter implies that $W^k(i)$ is a so-called root component. Increasing $\beta ^k_W$ only for root components is a criterion which may improve the computed lower bound. An example in which this is the case and a more detailed discussion is given for Wong’s dual ascent procedure [40].

In each iteration, an active terminal *i* and scenario *k* is chosen in Step 3 (chooseActiveTerminal). Next, the set $W^k(i)$ is computed by a reverse breadth-first search on $G^k_S$ and the maximum possible increase of $\beta ^k_W$ is computed and denoted by $\Delta$. Note that whenever the aforementioned condition $\tilde{c}^k_{ij} > \tilde{c}^k_{ji}$ holds for some edge $e=\{i,j\}\in \delta (W)$, then this amount must be subtracted when updating the reduced cost in Step 6. Terminals that have become inactive for the current *k* are removed from $T^k_a$ in Step 9 (removeInactiveTerminals). The procedure terminates as soon as no active terminals remain, in which case no variable $\beta ^k_W$ can be increased without violating some constraint. In Step 11, the dual multipliers $\varvec{\lambda }$ are computed based on each scenarios reduced cost. It holds that $c^k_e-\min \{\tilde{c}^k_{ij}, c^k_{ji}\}=\max \{\beta (\mathcal {W}^k_{ij}),\beta (\mathcal {W}^k_{ji})\}$ for each $e \in E$ and $k \in K$. Thus if $\varvec{\beta }$ is feasible to $(\text {SDC}^{D'}_3)$, the corresponding assignment $(\varvec{\beta },\varvec{\lambda })$ is feasible to $(\text {SDC}^D_3)$.

Further details (e.g., in which order to choose active terminals) follow closely dual ascent procedures for the STP (see, e.g., [28]), and are thus omitted. The following propositions state basic properties of Algorithm 1.

#### Proposition 3

$\bar{\varvec{\beta }}$ is feasible for $(\text {SDC}^{D'}_3)$ in each iteration of Algorithm 1.

#### Proof

The initial assignment of $$\tilde{\mathbf{c}}$$ implies $$\bar{\varvec{\beta }}=\mathbf {0}$$, which is feasible and $$ LB =0$$ corresponds to its objective value. In each iteration of the main loop, for the chosen terminal *i* and scenario *k*, and the resulting set *W*, it holds by definition that $$r \notin W$$ and $$\delta ^-(W)\cap A^k_S=\emptyset $$. It follows that $$\Delta > 0$$. At the beginning of any iteration, $$\beta ^k_W=0$$. As $$\Delta $$ is computed as the minimum feasible increase based on the reduced cost of all involved constraints, setting $$\beta ^k_W$$ to $$\Delta $$ results in feasible solution. $$\square $$

#### Proposition 4

The worst-case time complexity of Algorithm 1 is $\mathcal {O}(\sum \nolimits _{k \in K}|A|\min \{|A|,|T^k||V|\})$.

#### Proof

For each scenario $k \in K$ the following holds: In each iteration at least one arc will be saturated w.r.t. *k*. Moreover, for each $i \in T^k{\setminus }\{r\}$, at most |*V*| iterations can be performed, as at this point $r\in W^k(i)$, and *i* becomes inactive for *k*. Thus $\min \{|A|,|T^k||V|\}$ is an upper bound on the number of performed iterations for each $k \in K$. Each iteration takes at most |*A*| steps, as the most complex operation performed is a breadth-first search. $\square$

### Refinement by applying Benders decomposition

In this section a Benders decomposition based on $(\text {SDC}_3^{\text {FB}})$ is detailed. This approach is in the spirit of the two-stage B&C approach introduced in [2] for $(\text {SDC}_2)$. The *Benders master problem* is stated as follows. 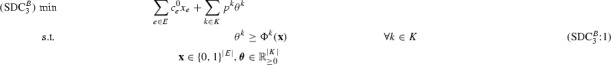


In this reformulation of $(\text {SDC}_3^{\text {FB}})$, the variables $\mathbf {z}$ and $\mathbf {w}$ associated to the second stage have been projected out of the model. In their place, non-negative variables $\varvec{\theta }$ denote the second-stage cost for each scenario. This property is ensured by constraints (SDC$^B_3$:1). For each $k \in K$ and first-stage solution $\bar{\mathbf{x}}$, the recourse function $\Phi ^k(\bar{\mathbf{x}})$ gives the corresponding second-stage cost, and is computed by solving the following *Benders subproblem*. 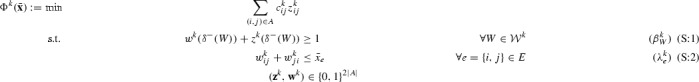


As the recourse function is neither convex or continuous, dynamically separated fractional and integral Benders optimality cuts are used in order to underestimate the value of $\Phi ^k(\bar{\mathbf{x}})$. In stochastic programming, this approach is also referred to as the L-shaped method (see, e.g., [3, 24] for problems with integer recourse). No Benders feasibility cuts are required as the SSTP has complete recourse.

Our implementation of this Benders decomposition approach is as follows: a branch-and-cut is employed to solve the master problem. Each time a new fractional master solution is found, Fractional Benders optimality cuts are added to the master. Each time an integer master solution is found, Integer Benders optimality cuts are inserted. Separation of these Benders cuts requires another cutting plane procedure (for separating fractional points) and a branch-and-cut procedure (for separating integer ones), which is why the method is called two-stage B&C (see [2, 27] for further details).

*Integer Benders optimality cuts*

Given $\bar{\mathbf{x}}$ integer, each Benders subproblem corresponds to an SAP in which the cost of arcs associated to the chosen first-stage edges are set to zero. Let $E^0_S=\{e \in E : \bar{x}_e=1\}$ denote the set of chosen first-stage edges induced by $\bar{\mathbf{x}}$. Then the following optimality cuts are valid. 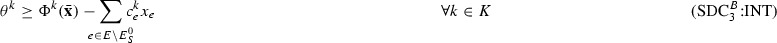


The validity of (SDC$^B_3$:INT) can be shown as follows. If an additional edge $e\notin E_S^0$ is added to the first stage, then the second-stage cost of scenario *k* can only decrease by at most $c^k_{e}$, i.e., the cost of the associated second-stage arc. Conversely, the cut remains valid if an edge $e\in E^0_S$ is removed from the first stage, as in this case the cost of each second-stage solution cannot decrease. In our implementation, we compute the value of $\Phi ^k(\bar{\mathbf{x}})$ by using the exact solver presented in [26].

*Fractional Benders optimality cuts*

For the purpose of cutting off fractional solutions $\bar{\mathbf{x}}$, we relax the integrality constraints of the Benders subproblem and dualize the resulting LP to obtain a valid underestimation of $\Phi ^k$, denoted by $\underline{\Phi }^k$. Variables $\varvec{\beta }$ and $\varvec{\lambda }$ are associated to constraints (S:1) and (S:2), respectively. 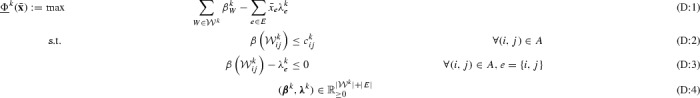 Let $(\bar{\varvec{\beta }}^k,\bar{\varvec{\lambda }}^k)$ denote an optimal solution to (D:1)–(D:4). Then the following optimality cuts are valid. 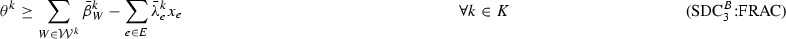


In our framework the computation of $(\bar{\varvec{\beta }}^k,\bar{\varvec{\lambda }}^k)$ is performed by applying row generation to the relaxed primal Benders subproblem. Thus constraints (S:1) are separated dynamically, following the two-stage B&C that has also been applied in [2, 27].

*Lagrangian Benders optimality cuts*

A large number of optimality cuts may form a potential bottleneck of the Benders decomposition approach. Especially at the beginning to the procedure, a large number of cutting planes may be separated due to little information on the original problem being presented in the master. A possible solution to this situation is the generation of an initial set of Benders optimality cuts using a set of high-quality dual solutions collected by the Lagrangian approach detailed in Sect. 3.1.

For this purpose, first observe that the Benders optimality cuts derived from suboptimal solutions to (D:1)–(D:1)–(D:4) are also valid. Consider for any $k \in K$ a feasible dual solution $(\bar{\varvec{\lambda }}^k,\bar{\varvec{\beta }}^k)$ to one of the relaxations of the Lagrangian subproblems $L^k(\bar{\varvec{\lambda }})$ detailed in Sect. 3.1. As the solution satisfies

$$\begin{aligned} \bar{\beta }\left( \mathcal {W}^k_{ij}\right) \le \min \left\{ \bar{\lambda }^k_{e},p^k c^k_{ij}\right\} \quad \forall (i,j)\in A, e=\{i,j\}, \end{aligned}$$

the scaled solution $(\bar{\varvec{\lambda }}^k,\frac{1}{p^k}\bar{\varvec{\beta }}^k)$ is feasible to (D:1)–(D:1)–(D:4). An alternative approach would be to move coefficients $p^k$ into the Benders subproblem by scaling each second-stage cost $c^k_{ij}$ appropriately. Note that this is however not recommendable due to potential numerical instabilities caused by small values of $p^k$.

### Overall framework

In this section we present further details of our algorithmic framework that combines the introduced approaches into an effective method. To efficiently attack large-scale instances, we focus on the development of a purely combinatorial Lagrangian heuristic framework. If the size of an instance allows, only in the refinement phase we invoke a state-of-the-art ILP solver. The main strategy is to initially apply “light-weight” methods, i.e., those of low worst-case runtime complexity. Thus we obtain iteratively improved primal and dual solutions, as well as their associated bounds. Using these, we can fix redundant parts of the instance early on (Sect. 3.1.1) before applying a more “heavy-weight” technique, i.e., the Benders decomposition approach detailed in Sect. 3.3.

With the aim to keep the Lagrangian relaxation approach from Sect. 3.1 “light-weight”, we resort to the approximation of subproblems, $L^k(\bar{\varvec{\lambda }})$, by fast primal and dual heuristics for the STP/SAP [8, 37, 40] in the employed subgradient optimization procedure. This is an attractive option, since for any $\bar{\varvec{\lambda }}, L^k(\bar{\varvec{\lambda }})$ corresponds to an SAP for each $k \in K$. In contrast, for the Benders decomposition approach, this is only the case if the master solution is integral in $\mathbf {x}$. Preliminary experiments have shown that such cases tend to occur rarely, and are solved exactly in our framework in order to guarantee termination of the B&B procedure. A pseudocode of the full framework is given by Algorithm 2. We proceed by explaining the used primal heuristics, and then discuss each phase of the framework separately.


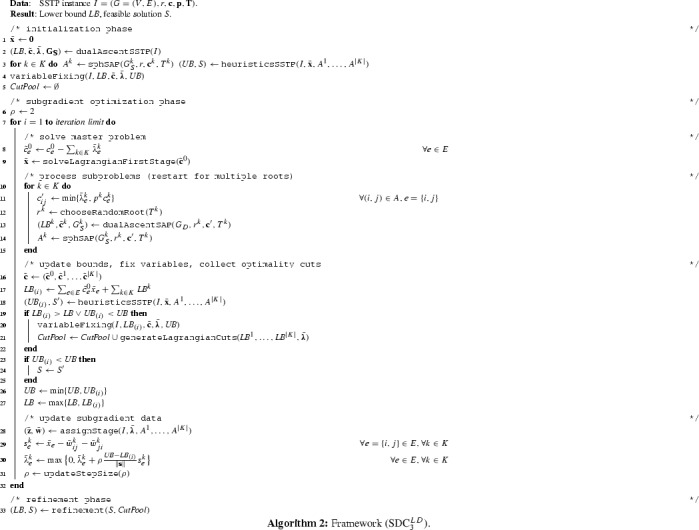


*Primal heuristics*

Two complementary procedures are used for obtaining high-quality primal solutions. In both of them solution construction is decomposed by stage.

- *First-stage-based heuristic* The first procedure exploits the fact that given a feasible first-stage solution $\bar{\mathbf{x}}$, the SSTP decomposes into |*K*| independent instances of the STP. This property is also exploited in other algorithms for the SSTP, namely the exact framework presented in [2] and the heuristic by Hokama et al. [19]. In the former, an exact algorithm is employed for the construction of second-stage solutions, while the latter uses a heuristic. In our implementation we follow the former and compute optimal solutions by using an exact algorithm. The solver proposed in [26] is applied with a short time limit to guarantee termination (five seconds per scenario in our implementation). In the Lagrangian approach, $\bar{\mathbf{x}}$ is obtained from the solution of $L^0(\bar{\varvec{\lambda }})$. In the Benders approach, $\bar{\mathbf{x}}$ is obtained by rounding fractional master solutions, i.e., for each edge $e\in E$, if $\bar{x}_e\ge 0.5$, the variable is set to one, otherwise to zero. This scheme is potentially time-consuming, as even after fixing the first stage |*K*| instances of an NP-hard problem remain to be solved. Thus the next scheme details a computationally cheaper approach.
- *Second-stage-based heuristic* In the second construction procedure, we exploit the fact that in the employed subgradient optimization algorithm, for each $k \in K$, a heuristic Steiner arborescence $A^k\subseteq A$ is constructed as solution to $L^k(\bar{\varvec{\lambda }})$. From these Steiner arborescences, an optimal allocation between the first and second stage can be computed as follows: For each $e=\{i,j\}\in E$, set $x_e=1$ if $\sum \nolimits _{k \in K : (i,j) \in A^k\vee (j,i)\in A^k} c^k_{e} \ge c^0_e$, otherwise, set $x_e=0$. The assignment of $\mathbf {z}$ and $\mathbf {w}$ can then be performed accordingly.

The consecutive execution of both procedures in our framework is denoted by $\texttt {heuristicsSSTP}$. Input parameters are a (potentially fractional) first-stage vector $\mathbf {x}$, as well as a set of second-stage Steiner arborescences $(A^0,\dots ,A^{|K|})$.

*Additional variable fixing*

The STP offers a large arsenal of reduction tests, which allow the transformation of a problem instance into a smaller instance if specific conditions are satisfied. The simplest transformation corresponds to the elimination of edges. The following two simple tests can be easily translated from their STP counterparts [7] into fixing variables of $(\text {SDC}_3^{\text {FB}})$. Note that the same does not hold for $(\text {SDC}_2^{\text {FB}})$, since as already stated, in this case the second-stage solutions do not necessarily form Steiner arborescences. In order to benefit from these tests (that are valid on undirected graphs) second-stage arcs are only fixed to zero if both $z_{ij}^k$ and $z_{ji}^k$ can be fixed to zero.

The following definitions are required: For each $k \in K$, let $G^k_\mathbf {z}=(V,E_\mathbf {z})$ denote the undirected second-stage graph induced by variables $\mathbf {z}^k$ that have not been fixed to zero, i.e., $E_\mathbf {z}:=\{e=\{i,j\} \in E : z^k_{ij}\ne 0 {\vee } z^k_{ji}\ne 0\}$. Let the set $\mathcal {P}^k_{ij}$ denote all paths that connect *i* and *j* on $G^k_{\mathbf {z}}$. A path is *elementary* if and only if its endpoints are terminals from $T^k$. Let the *bottleneck Steiner distance* relative to scenario *k* be defined as

$$\begin{aligned} SD^k_{e}:= & {} \min \Big \{\max \Big \{\sum _{e \in P_S}c^k_{e} : P_S \text { is an elementary subpath of } P\Big \} : P \in \mathcal {P}^k_{ij}\Big \}\\&\quad \forall e=\{i,j\}\in E^k \end{aligned}$$

The concept of the bottleneck Steiner distance has been first considered in [7], and allows the formulation of an effective edge elimination test, which has been employed in several successful algorithms for the STP [8, 10, 31]. Using these definitions, the following two rules for fixing variables are stated for the SSTP and formulation $(\text {SDC}_3^{\text {FB}})$. These rules are always applied after the ones based on reduced cost detailed in Sect. 3.1.1.

- *Second-stage degree one (D1).* If a non-terminal $i \in V{\setminus } T^k$ has only one adjacent edge $e=\{i,j\}$ on $G^k_\mathbf {z}$, then $w^k_{ij}, w^k_{ij}, z^k_{ij}$, and $z^k_{ji}$ can be fixed to zero. This can be done, as these arcs would never be part of any Steiner arborescence spanning $T^k$.
- *Second-stage bottleneck Steiner distance (SD).* If $SD^k_{e} < c^k_{e}, e \in E$, then $z^k_{ij}$ and $z^k_{ji}$ can be fixed to zero. Concerning the validity of this condition, consider the SD test for the STP [7]. The test states that any edge $e\in E$ with edge cost $c_e$ greater than its bottleneck Steiner distance cannot be part of an optimal Steiner tree. For the SSTP, note that once the set of optimal first-stage edges $E^0_S$ is known, the SSTP decomposes into |*K*| independent instances of the STP. In each of these instances, $T^k$ is the set of terminals, and edge costs $\bar{c}^k_{e}=c^k_e$ if $e \notin E^k_S$ and $\bar{c}^k_{e}=0$ otherwise. For each edge $e \in E$, let $\overline{SD}^k_e$ denote the bottleneck Steiner distances on each of these instances, i.e., computed based on the modified edge cost $\bar{\varvec{c}}^k$. The SD test is valid given an optimal set $E^0_S$. Since $\overline{SD}^k_e< SD^k_e$ of each $k\in K$ and $e \in E$ the test is valid for the SSTP.
  In our implementation, we compute a heuristic approximation of $SD^k_e$ for edges $e\in E$ by a modified implementation of Dijkstra’s algorithm, in which additional distance labels are stored which are set to zero whenever a terminal is encountered. Moreover, due to the test condition, the search can be restricted to paths of length at most $c^k_{e}$.

Algorithm 2 lists the pseudocode of the proposed framework. Three phases can be distinguished: (i) initialization by dual ascent, (ii) subgradient optimization, and (iii) refinement by using the Benders decomposition approach.

- *Initialization (Dual ascent)* In Step 2, the dual ascent procedure for the SSTP (cf. Sect. 3.2, denoted by dualAscentSSTP in the pseudocode) is used to obtain a globally valid lower bound $LB$, associated reduced cost $\tilde{\mathbf{c}}$, and an initial set of dual multipliers $\bar{\varvec{\lambda }}$. Moreover, the corresponding saturation graphs $G^k_S$ are returned. For each $k\in K$, the shortest path heuristic for the STP [37] (sphSTP) is applied on $G^k_S$ in order to obtain a heuristic Steiner arborescence $A^k$. Running the heuristic on $G^k_S$ instead of $G_D$ is known to improve the performance of STP heuristics due to complementary slackness [31]. Naturally, the same holds in case of the SSTP. Based on an initial first-stage solution ($\bar{\mathbf{x}}=\mathbf {0}$) and the computed Steiner arborescences, a feasible solution *S* to the SSTP is computed in Step 4 (heuristicsSSTP). Throughout the algorithm, *S* represents the incumbent solution. After this initial computation of lower and upper bounds, an initial round of variable fixing is applied in Step 5 (cf. Sect. 3.1.1, variableFixing). The set *CutPool* is used to collect optimality cuts derived from dual solutions in the next phase.
- *Subgradient optimization* Parameter $\rho$ denotes the step-size used for updating the Lagrangian dual multipliers $\bar{\varvec{\lambda }}$ in each iteration. Initially, $\rho =2$. Further details on the employed schedule for updating $\rho$ are given after all other operations executed within each iteration have been presented. The main loop is executed until a fixed iteration limit has been reached, or all first-stage variables have been fixed to one or zero. In the latter case, the SSTP decomposes into a set of STP instances, which are solved using the exact solver from [26] that is also applied in the primal heuristic (but here without the timelimit).
  In Steps 9–10, the Lagrangian costs are computed and the Lagrangian subproblem $L^0(\bar{\varvec{\lambda }})$ is solved (solveLagrangianFirstStage). At this point we exploit a simple observation, namely that in an optimal solution the subgraph induced by the first-stage, i.e., $G[E^0_S]$, is cycle-free.
  This property is efficiently incorporated into the solution of $L^0(\bar{\varvec{\lambda }})$: Variables $\mathbf {x}$ with non-positive Lagrangian cost are set to one iteratively in ascending order according to $\tilde{\mathbf{c}}$. Any variable that would introduce a cycle in the induced subgraph is skipped.
  In Steps 11–16, for each $k \in K$, the Lagrangian subproblem $L^k(\bar{\varvec{\lambda }})$ is solved heuristically. This requires the computation of a lower bound $LB^k$ and a heuristic primal solution. Note that the latter is required in order to compute a subgradient for updating $\bar{\varvec{\lambda }}$. In Step 12, the modified cost vector $\mathbf {c'}$ is computed as the minimum between $\bar{\varvec{\lambda }}$ and $p^k \mathbf {c}^k_e$. In the next steps, the dual ascent procedure for the SAP [40] (dualAscentSAP) and the shortest path heuristic [37] (sphSAP) are executed based on a randomly selected root node $r^k$, terminals $T^k$, and the modified cost $\mathbf {c'}$. As in the initialization phase, the saturation graph $G^k_S$ is exploited to improve the performance of the primal heuristic. The random choice of the root node perturbs both the primal and dual heuristic, and as a consequence repeating Steps 11–16 for multiple roots may yield solutions of improved quality. In our implementation, we repeat these steps at most five times, and keep only the best primal and dual solutions.
  If for one scenario $k\in K$, the solutions’ corresponding lower and upper bounds coincide, then of course no further repetitions are necessary for this scenario, as this subproblem is solved to optimality. We choose to not represent this repetition in the pseudocode for brevity and ease of exposition.
  In Steps 17–28, the computed information is aggregated (reduced cost, bounds, and Steiner arborescences). The primal heuristic is started in Step 19 (heuristicsSSTP). Due to its computational complexity, the first-stage-based heuristic (which requires the solution of STP instances) is only applied every tenth subgradient iteration, or to refine the solution computed by the second-stage-based heuristic, if the constructed solution exceeds the quality of the incumbent solution *S*. The variable fixing in Step 21 (variableFixing) is only executed if the global lower or upper bound improved during the current iteration. Moreover, in this case |*K*| optimality cuts (generateLagrangianCuts) are generated and added to the *CutPool*. Note that for the generation of the cuts, it is sufficient to use the lower bounds for each subproblem – the concrete assignment of dual variable $\varvec{\beta }$ is not required.
  Finally, in Steps 29–32, a subgradient $\mathbf {s}$ is computed based on the primal solutions to subproblems $L^k(\bar{\varvec{\lambda }}), k \in K,$ computed in the course of this iteration. For this purpose, in Step 29 the corresponding vectors $\bar{\mathbf{z}}$ and $\bar{\mathbf{w}}$ are retrieved (assignStage) as follows: For each $k\in K, z^k_{ij}=w^k_{ij}=0$ if $(i,j)\notin A^k$. For $(i,j) \in A^k, w^k_{ij}=1$ and $z^k_{ij}=0$ if $\lambda ^k_e < p^k c^k_e$. Otherwise, the converse holds.
  The dual multipliers $\bar{\varvec{\lambda }}$ are updated based on the standard scheme for subgradient optimization (see, e.g., [9, 18]), as is the step-size $\rho$. More complex update schedules have been explored in preliminary experiments, but as no significant improvements in convergence could be achieved on the tested benchmark set, we prefer the standard scheme due to its simplicity: If for $\kappa$ consecutive iterations the global lower bound $LB$ does not improve, $\rho$ is halved. We set $\kappa :=20$ and the iteration limit to 250.
- *Refinement (Benders decomposition)* In Step 34, the two-stage B&C based on Benders decomposition is started (refinement). We initialize this final step using the incumbent solution *S* and the Lagrangian optimality cuts collected in *CutPool*. Similarly, we initialize the Benders subproblems using a set of connectivity cuts, which we derived from the SAP dual ascent algorithm (see, e.g., [31]).
  Moreover, whenever a subproblem is solved, all separated connectivity cuts remain valid for any given first-stage vector. Thus these inequalities are also used to initialize all subsequent iterations. In addition to the separation of connectivity cuts, we also chose to separate the strengthening flow-balance inequalities (SDC$_3$:FB) dynamically. A related family of valid inequalities can be derived from the SAP [21]. For the SAP, these inequalities are known to be implied by the optimal solution of the LP relaxation of its directed cut formulation. However, it is known empirically that a subset of them (dynamically separated) can decrease the number of necessary cutting plane iterations. The inequalities (SDC$_3$:4) are valid for $(\text {SDC}_3^{\text {FB}})$, and thus also for $(\text {SDC}_3)$. 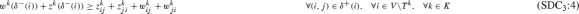 Tailing-off behavior of the cut-loop at the root node is detected as follows. Let $$ LB ^B_{(i)}$$ denote the lower bound at iteration *i* of the row generation procedure applied to $$(\text {SDC}^B_3)$$ within the root node of the B&C tree. If for $$\kappa '$$ consecutive iterations the relative lower bound improvement, i.e., ($$ LB ^B_{(i+1)}- LB ^B_{(i)})/ LB ^B_{(i+1)}$$, remains below a threshold $$\tau $$, then the cut loop is terminated and we begin to branch. In our implementation $$\kappa ':=5$$ and $$\tau :=1e-10$$. The same approach is applied within each subproblem.

## Computational results

All algorithms have been implemented in C$++$. Recall that the subgradient procedure is a combinatorial approach, and that only for the Benders decomposition part, CPLEX 12.7 is used as a ILP solver. Tests have been performed single-threaded on an Intel Xeon CPU E5-2670v2 (2.5 GHz). On each test run a time limit of 1 h and a memory limit of 6 GB is set. The performance evaluation is conducted on instances from the SSTPLib [34], which are part of the benchmark set employed during the 11th DIMACS implementation challenge on Steiner trees [19, 35]. These instances have been generated from STP instances available in the SteinLib [2, 22]. Moreover, based on the same scheme we have generated new large-scale benchmark instances from real-world STP instances [25] which have also been used during the challenge. The graphs in these instances have been generated from spatial data for the design of infrastructure networks. We restrict ourselves to the ten smallest instances from this dataset (“vienna-i-simple”), which already exceed the size of previous instances significantly. The new dataset is referred to as VIENNA and made available online at https://msinnl.github.io/pages/sstp.html (detailed results for each instance are also availabe). The average characteristics of all benchmark instances are listed in Table 1.

For each dataset except VIENNA, $|K|\in \{5,10,20,50,75,100, 150,200,250,300$, $400,500,750,1000\}$. For VIENNA, only instances with up to 50 scenarios have been created due to their large size. For performance plots instances are aggregated into a group of small instances $\text {SMALL}:=\{\text {K100}, \text {P100}, \text {LIN01-10}, \text {WRP}\}$ and a group of large-scale instances $\text {LARGE}:=\{\text {VIENNA}\}$. Tests are conducted on 600 benchmark instances in total.

**Table 1:** Basic properties of our benchmark instances

Dataset	Inst [#]	|*V*|			|*E*|			|*K*|		
		Min	Avg	Max	Min	Avg	Max	Min	Avg	Max
K100	154	22	31	45	64	115	191	5	272	1000
P100	70	66	77	91	163	194	237	5	272	1000
LIN01-10	140	53	190	321	80	318	540	5	272	1000
WRP	196	10	194	311	149	363	613	5	272	1000
VIENNA	40	1991	5756	9574	3176	9347	16,208	5	21	50

In each table reported in the remainder of this section, columns *t* [s] and $t_b$ [s] denote the running time and the time at which the best solution has been found in seconds. Columns *inst*[#] and *solv*[#] denote the number of instances in a given group and the number of instances solved to optimality, respectively. Columns *g* [%] and *Pg* [%] denote the relative optimality and primal gap, computed as *g* [%]$:=(\text {{ UB}}-\text {{ LB}})/\text {{ UB}}$ and *Pg* [%]$:=(\text {{ UB}}- BEST )/\text {{ UB}}$, respectively. Here the values $LB$ and $UB$ refer to the best lower and upper bound computed by a method on a given instance, while $BEST$ denotes the best upper bound computed by one of the compared methods.

We begin our computational study by analyzing the contributions of the proposed components of the Lagrangian decomposition framework. Once their effectiveness has been established, we compare the performance of the total framework with state-of-the-art exact and heuristic solution methods given in [2, 19, 27], respectively.

### Effects of the dual ascent initialization

We first analyze the benefits of initializing the subgradient algorithm (see Sect. 3.4) with the dual solution computed by the dual ascent procedure (see Sect. 3.2). For this purpose, both the variable fixing based on reduced cost and reduction tests (see Sects. 3.1.1 and 3.4), as well as the refinement (see Sect. 3.3) have been deactivated.

The following two settings are compared:

- L: This is a basic subgradient procedure in which an initial set of multipliers is computed as $\lambda ^k_e=p^kc^k_e, e \in E, k \in K$, which corresponds to no first-stage edge being selected. In this setting the SSTP dual ascent is not executed during the initialization phase, the initial primal solution *S* is computed only using the first-stage-based heuristic (i.e., Step 3 of Algorithm 2 is hence skipped due to no saturation graphs being available).
- DL: In this setting the SSTP dual ascent is executed as specified in the overall framework (Algorithm 2) and the the subgradient algorithm is started from the computed dual multipliers.

**Fig. 8:**
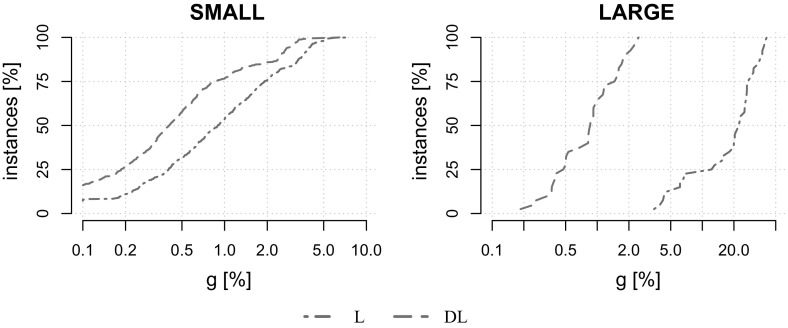
Optimality gap charts for SMALL and LARGE instances with dual ascent initialization of the subgradient algorithm (DL) and without (L)

Figure 8 reports the optimality gaps obtained by the two settings after the 1-h time limit. In the two cumulative charts (one per group), we report the percentage of instances solved within a certain optimality gap. One observes that this warm-starting has a significant impact on the performance of the subgradient procedure, both on small and large-scale instances. The effect is particularly pronounced for group LARGE, where the worst optimality gaps remain below to 3%. On the contrary, if the SSTP dual ascent is skipped, the final gap achieved by the subgradient method lies between 5 and 30% for almost all instances of this group. This significant optimality gap is a direct consequence of low-quality dual solutions, which yield lower bounds far from the optimum. As a consequence, also the upper bounds rarely improve beyond the ones computed by the initial heuristic, further amplifying the difference between the tested settings. When starting from the given dual ascent solution, improvements of lower and upper bounds have usually been observed after few iterations on almost all benchmark instances. Thus these dual multipliers are a good starting point for further improvement.

**Table 2:** Effects of variable fixing

Dataset	DR					DLR				
	Fixed [%]	$f_1$ [%]	$f_2$ [%]	*g* [%]	*t* [s]	Fixed [%]	$f_1$ [%]	$f_2$ [%]	*g* [%]	*t* [s]
K	35	21	14	2.55	0	71	57	15	0.29	11
P	48	33	15	1.80	1	82	63	18	0.06	18
LIN01-10	10	9	1	3.60	8	27	24	3	1.81	256
WRP	17	9	8	2.04	9	18	10	8	0.32	222
VIENNA	15	5	10	2.02	154	20	9	11	0.88	1782

### Effects of variable fixing

In order to quantify the effects and benefits of the variable fixing conditions presented in Sects. 3.1.1 and 3.4, we first report on the number of variables/edges that can be eliminated due to these tests. Observe that these operations are valid for any given vector of dual multipliers. Since two algorithms have been proposed for deriving strong dual multipliers (namely, the dual ascent and the subgradient procedure), we investigate the effects of variable fixing on the following two settings:

- DR: This is the proposed SSTP dual ascent algorithm enhanced by variable fixing. This means that only the initialization phase of the proposed framework is executed, i.e., Algorithm 2 is executed up to Step 5.
- DLR: This is the DL setting from the previous section, enhanced by the variable fixing. In other words, Algorithm 2 is executed up to Step 33, and the refinement phase is omitted.

Table 2 reports the results of this comparison. Column *fixed*[%] lists the average percentage of eliminated variables. In the following, we use $f_1$ and $f_2$ in order to refer to the two types of variable fixing rules introduced in this article: reduced-cost-based fixing (Sect. 3.1.1) and reduction-test-based fixing (Sect. 3.4), respectively. Columns $f_1$ [%] and $f_2$ [%] report the corresponding average percentages of eliminated variables by the respective rules. In addition, we report the average running time and optimality gaps of the two settings.

We observe that already the dual ascent procedure for the SSTP provides very useful primal and dual bounds that allow a sizable portion of variables (up to almost 50%) to be eliminated. The corresponding optimality gaps are within few percent, explaining the effectiveness of $f_1$, which profits from the availability of tight bounds. However, the effects achieved by $f_2$ are also considerable, as they manage to eliminate up to 15% of all variables. Moreover, on dataset VIENNA these tests manage to even outperform $f_1$. Even in the worst case, already after the initialization phase at least 10% of all variables can be eliminated on average.

**Fig. 9:**
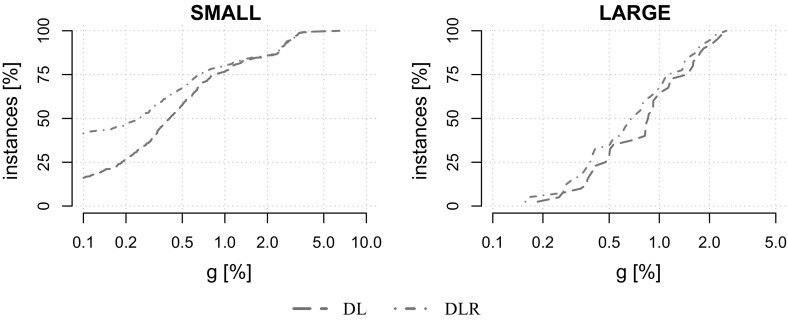
Optimality gap charts for SMALL and LARGE with (DLR) and without (DL) reduction tests and variable fixing

The effectiveness of the variable fixing is further amplified during the subgradient procedure. Furthermore, the framework manages to decrease the remaining optimality gap to less than 2% on average. The most impressive results are achieved on dataset P100, where on average 82% of all variables are eliminated. Even on dataset LIN01-10, on which the largest average optimality gap remains (1.81%), almost 30% of all variables can be eliminated. Between the initialization and subgradient phase, the amount of variables fixed by $f_1$ is more than doubled in most cases. The only dataset seemingly resistant to the bound-based variable fixing $f_1$ is WRP, on which even after reducing the gap from over 2% to almost 0.3% during the subgradient phase, only few additional variables can be fixed.

Figure 9 demonstrates the influence of the employed variable fixing w.r.t. the overall performance. We compare the optimality gaps at the end of the subgradient procedure without fixing (setting DL described above), and with fixing enabled (setting DLR). The results on SMALL instances show that the proposed tests are effective. The tighter the bounds, the larger the decrease in the optimality gap. More precisely, for about 20% of SMALL instances, the gap without variable fixing is below 0.1%. After including the variable fixing, 40% of SMALL instances can be solved within 0.1% to optimality. On LARGE instances, an improvement can still be observed, but the bounds appear to be not sufficiently tight in order to yield a significant boost of performance. However, even though the effects of the proposed fixing on the subgradient algorithm may appear minor, such eliminations are certainly essential when entering the exact algorithm during the refinement phase.

### Effects of the Benders decomposition approach

**Fig. 10:**
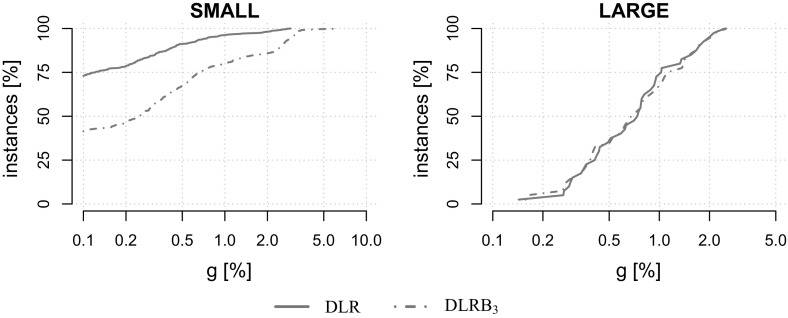
Optimality gap charts for SMALL and LARGE with (DLRB$_3$) and without (DLR) Benders decomposition applied as a refinement procedure

Upon the termination of the subgradient phase, our framework enters the refinement phase, in which the Benders decomposition approach is called. We now report on the improvements that are achieved in this final phase. Note that we still only consider the performance of the method in the root node of the B&C tree. We compare the best performing setting so-far (DLR) with the setting in which the Benders decomposition approach is called after the subgradient algorithm (denoted by DLRB$_3$). Figure 10 reports the optimality gaps of the two settings. As expected, an improvement of the computed bounds is achieved. The results are particularly striking for the SMALL instances. On the LARGE instances, the effect is much less pronounced, as these instances appear to be too large to be effectively handled by using an ILP solver.

**Fig. 11:**
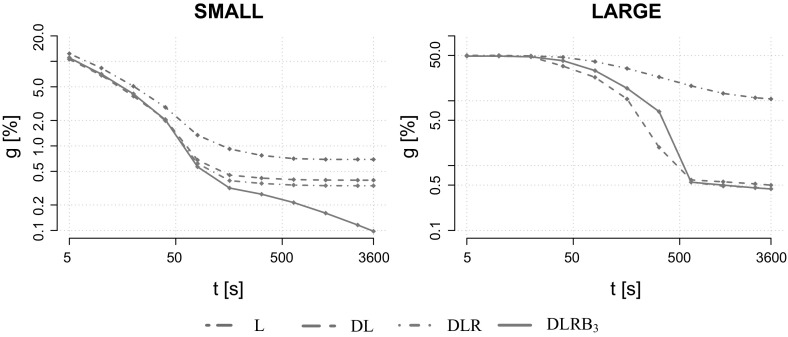
Average development of the optimality gap w.r.t. running time

Finally, Fig. 11 shows the average development of the optimality gap relative to running time. The colors and line styles are chosen according to the configurations applied in the previous Sections (see Figs. 8, 9, 10). Note that a time limit of 1 h is applied. If an instance finishes processing the root B&B node early, its final gap is used in the computation of the average gap for subsequent time points. The average gap is only computed at the time instants shown by the dots on each line while linear interpolation is applied in between. On SMALL and LARGE instances, we can observe that the convergence is significantly improved by starting from the solution provided by the SSTP dual ascent procedure. Moreover, one can observe that on the LARGE instances, although the variable fixing finally pays off in the form of a smaller final optimality gap, initially it can be a computational burden if the available bounds are not sufficiently tight. In our implementation, a potential bottleneck is the computation of special distances, which are recomputed from scratch in each check. Thus this effect could be remedied by an improved implementation in which these distances are updated dynamically [31].

### Comparison with the state-of-the-art

The results obtained in the previous sections suggest that the combination of our framework’s components yields a computational performance which is fairly robust both with respect to small and large-scale instances. In this section, we proceed by comparing the performance of our new method to the state-of-the-art exact algorithm presented in [2, 27], a Benders decomposition approach based on a two-stage B&C and formulation $(\text {SDC}_2)$. Moreover, the quality of the obtained primal solutions is compared with the heuristic by Hokama et al. [19], a genetic algorithm introduced during the 11th DIMACS implementation challenge on Steiner trees.

In order to provide a fair comparison, the method from [2, 27] has been carefully reimplemented. Moreover, the approach has been improved as follows: The strengthening flow-balance inequalities introduced in Sect. 2.2 are separated dynamically, together with the following valid inequalities, which are the counterpart to (SDC_3_:4) for formulation $(\text {SDC}_2)$. 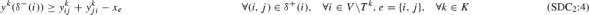


For each $k\in K$, the cut pool of the corresponding subproblem is initialized using the set of connectivity cuts detected by the dual ascent procedure for the SAP. The resulting algorithmic approach is denoted by B$_2$. In the following experiments, the performances of DLRB$_3$ and B$_2$ are compared with respect to the exact solution of instances, i.e., the ILP solver is not restricted anymore to the root node of the B&C tree as in the previous section.

**Fig. 12:**
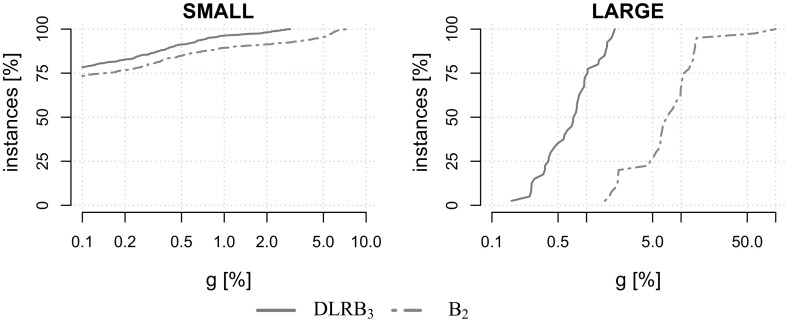
Optimality gap charts comparing DLRB$_3$ and B$_2$

Figure 12 shows optimality gap charts on instance groups SMALL and LARGE. On the LARGE instances, DLRB$_3$ significantly outperforms B$_2$ with respect to the computed optimality gaps. The results on SMALL instances show that DLRB$_3$ manages to obtain significantly smaller gaps in the worst-case (3% instead of 7%). In addition, also more instances can be solved within a gap of 0.1% than with B$_2$.

A more detailed view of these results is given in Tables 3, 4, 5 and 6. In each table instances have been aggregated based on |*K*|. Moreover, the average quality of primal solutions (columns *Pg* [%]) and the average times at which the best solutions have been found (columns $t_b$ [s]) are reported. The results of the heuristic by Hokama et al. [19] are denoted by H. Their results have been computed on an Intel Xeon CPU E3-1230 V2, (3.30 GHz), implemented in C$++$, and run for a time limit of 1 h. The CPU is thus slightly faster than the one used for testing our own implementations.

Table 3 summarizes results on dataset K100 and P100. Both DLRB$_3$ and B$_2$ manage to solve these instances to optimality within the time limit. On K100, both methods require approximately equal time for this task, while DLRB$_3$ usually obtains the best primal solutions faster, due to its “light-weight” initialization approach. On P100, DLRB$_3$ significantly outperforms B$_2$. On both K100 and P100, the solution quality obtained by H is on average within 1%. However, the time required to reach this quality is significantly higher than the time needed to solve the instance to optimality by DLRB$_3$.

**Table 3:** Results on datasets K100 and P100 (all solved to optimality by DLRB$_3$ and B$_2$, columns *Pg* [%] are thus omitted)

|*K*|	*t* [s]		*Pg* [%]	$$t_b$$ [s]			|*K*|	*t* [s]		*Pg* [%]	$$t_b$$ [s]		
	DLRB$_3$	B$_2$	H	DLRB$_3$	B$_2$	H		DLRB$_3$	B$_2$	H	DLRB$_3$	B$_2$	H
5	1	1	2.31	**0**	1	1	5	**0**	13	0.71	**0**	12	3
10	1	1	0.86	1	1	1	10	**1**	12	0.84	**0**	9	5
20	2	2	0.68	1	1	2	20	**2**	15	0.98	**1**	9	13
50	3	3	0.81	2	2	5	50	**2**	32	0.83	**1**	21	32
75	4	5	0.55	**2**	4	8	75	**6**	43	1.03	**3**	30	48
100	5	5	0.58	**3**	4	11	100	**8**	58	1.05	**1**	18	59
150	9	**8**	0.57	6	6	16	150	**13**	81	0.77	**2**	26	105
200	13	**12**	0.52	**8**	9	23	200	**20**	110	0.94	**3**	32	139
250	**15**	16	0.55	**6**	11	28	250	**26**	145	0.83	**4**	37	177
300	19	**17**	0.88	**9**	14	30	300	**29**	159	0.77	**6**	44	184
400	27	**22**	0.72	**15**	18	40	400	**41**	206	0.89	**4**	55	272
500	32	**28**	0.60	18	18	57	500	**49**	270	0.89	**7**	65	340
750	**44**	47	0.66	**26**	36	93	750	**83**	419	0.81	**28**	129	534
1000	68	**61**	0.82	**32**	35	121	1000	**107**	536	1.00	**10**	156	689

Bold values indicate the best obtained values

Table 4 summarizes the results on dataset LIN01-10 and WRP. We observe that on LIN01-10, DLRB$_3$ significantly outperforms the results achieved by $B_2$. This is the case with respect to the number of instances solved, average remaining optimality gap, the average required running time, and the quality of primal solutions. Moreover, the heuristic approach is outperformed by both DLRB$_3$ and B$_2$.

On dataset WRP, DLRB$_3$ computes slightly better bounds on average and also better primal solution. However, the average solution time is slightly higher than $B_2$, and as a consequence in some cases less instances can be solved to optimality within the time limit. This property is mainly a consequence of the larger number of variables of model $(\text {SDC}_3^{\text {FB}})$, a burden that outweights its gain on this specific type of dataset. As already noted in Sect. 4.2, despite the availability of very tight bounds, our reduced-cost-based variable fixing tests are not capable of eliminating many variables on these instances. Here, a more light-weight modeling approach appears more beneficial when the main aim is to solve instances to optimality. Finally, we can observe that the heuristic approach H manages to outperform both DLRB$_3$ and B$_2$ with respect to solution quality on instances with at least $|K|=200$. However, on average a significant amount of time is required to reach this quality.

**Table 4:** Results on datasets LIN01-10 (10 instances per |*K*|) and WRP (14 instances per |*K*|)

Dataset	|*K*|	Solv[#]		*g* [%]		*t* [s]		*Pg* [%]			$t_b$ [s]		
		DLRB$_3$	B$_2$	DLRB$_3$	B$_2$	DLRB$_3$	B$_2$	DLRB$_3$	B$_2$	H	DLRB$_3$	B$_2$	H
LIN01-10	5	8	6	**0**.**35**	0.91	**1294**	1470	**0**.**00**	0.24	1.26	994	725	16
	10	8	6	**0**.**06**	0.65	**1213**	1478	**0**.**00**	0.24	1.14	793	727	27
	20	8	6	**0**.**05**	0.51	**1041**	1489	**0**.**00**	0.13	0.77	889	27	62
	50	9	6	**0**.**00**	1.01	**1155**	1542	**0**.**00**	0.07	0.57	903	96	146
	75	9	6	**0**.**00**	1.32	**1319**	1583	**0**.**00**	0.06	0.67	1147	131	187
	100	8	6	**0**.**03**	1.52	**1426**	1603	**0**.**00**	0.06	0.72	1322	159	260
	150	6	6	**0**.**11**	1.76	**1533**	1656	**0**.**00**	0.03	0.62	673	150	355
	200	6	6	**0**.**22**	1.98	**1557**	1717	**0**.**00**	**0**.**00**	0.54	86	196	684
	250	6	6	**0**.**32**	2.15	**1592**	1773	**0**.**00**	0.01	0.53	466	235	708
	300	6	6	**0**.**39**	2.21	**1636**	1839	**0**.**00**	**0**.**00**	0.64	131	288	947
	400	6	6	**0**.**67**	2.28	**1690**	1986	**0**.**00**	**0**.**00**	0.59	166	389	1223
	500	6	6	**0**.**83**	2.35	**1742**	2120	**0**.**00**	**0**.**00**	0.53	213	484	1659
	750	6	5	**0**.**97**	2.53	**1900**	2458	**0**.**00**	**0**.**00**	0.51	321	694	2407
	1000	6	3	**1**.**01**	2.85	**2065**	2543	**0**.**00**	0.10	0.50	562	1061	3072
WRP	5	6	6	**0**.**10**	0.16	**2126**	2177	**0**.**00**	0.04	0.23	821	1270	14
	10	7	6	**0**.**08**	0.18	**1894**	2143	**0**.**00**	0.07	0.18	881	1352	30
	20	7	7	**0**.**08**	0.15	**1866**	2023	**0**.**01**	0.06	0.13	1098	1541	73
	50	7	7	**0**.**09**	0.12	**1950**	1974	**0**.**02**	0.04	0.11	1679	1610	170
	75	6	7	**0**.**09**	0.16	2184	**2108**	**0**.**01**	0.06	0.11	1525	1717	277
	100	7	7	**0**.**11**	0.12	**2107**	2152	**0**.**04**	0.03	0.10	1372	1740	321
	150	7	7	**0**.**14**	0.17	2184	**2158**	**0**.**06**	0.08	0.08	1510	1841	494
	200	6	7	**0**.**16**	0.22	2400	**2263**	**0**.**05**	0.10	**0**.**05**	1561	1567	**627**
	250	5	6	**0**.**17**	0.23	2670	**2424**	0.06	0.10	**0**.**05**	1473	1810	745
	300	6	6	**0**.**15**	0.26	2760	**2477**	**0**.**04**	0.12	0.05	1762	2012	1111
	400	6	6	**0**.**19**	0.29	2874	**2526**	0.05	0.13	**0**.**04**	1370	2235	1381
	500	5	5	**0**.**24**	0.31	2983	**2736**	0.07	0.14	**0**.**03**	1194	2501	1995
	750	3	5	0.39	0.39	3120	**2966**	0.18	0.20	**0**.**03**	1016	2199	2233
	1000	3	4	**0**.**37**	0.47	3323	**3132**	0.14	0.24	**0**.**01**	891	2518	4070

Bold values indicate the best obtained values

Table 6 reports results on dataset VIENNA. As this large-scale dataset has been newly introduced in this article with the aim of further testing the limits of our methods, unfortunately no results for the heuristic by Hokama et al. are available. We can observe that indeed, these instances are more challenging than the previously existing datasets, as no instance could be solved to optimality by any of the tested methods. The obtained results show that on average DLRB$_3$ significantly outperforms B$_2$ both with respect to optimality gaps and the quality of primal solutions. Moreover, the main difference between these two methods lies mostly in the quality of lower bounds, which for DLRB$_3$ are much tighter than for B$_2$. This behavior is mainly due to the superior scalability of our Lagrangian heuristic. As a consequence, even instances with up to 50 scenarios can be handled effectively. In this latter case, average gaps obtained by B$_2$ are as high as 22%, whereas the respective gaps obtained by DLRB$_3$ remain below 1%.

The success of our approach can be partially attributed to the improved quality of lower bounds due to the flow-balance inequalities (SDC$_3$:FB). In our algorithmic framework, DLRB$_3$, these constraints are exploited in the refinement phase, where they are explicitly added to the Benders subproblems. To measure the impact of these constraints on practical solving, we provide a comparison of lower bounds for two formulations. Table 5 compares the quality of lower bounds at the root node of our DLRB$_3$ framework, which corresponds to the $(\text {SDC}_3)$ formulation enhanced by the inequalities (SDC$_3$:FB) (denoted by $(\text {SDC}_3^{\text {FB}})$), and those obtained by the B$_2$ framework, which corresponds to the previous state-of-the-art formulation $(\text {SDC}_2)$, enhanced by the weaker (SDC$_2$:FB) constraints (denoted by $(\text {SDC}_2^{\text {FB}})$). Recall that the two basic formulations, $(\text {SDC}_2)$ and $(\text {SDC}_3)$ are equally strong, and that the bounds obtained by $(\text {SDC}_2^{\text {FB}})$ are in theory stronger, whereas those obtained by $(\text {SDC}_3^{\text {FB}})$ are the strongest ones (cf. Fig. 6). For the two groups, SMALL and LARGE, and for each of the two formulations, the following values are reported in Table 5: the average gap at the root node of the branch-and-bound tree (root gap [%]), and the average number of branch-and-bound nodes (# nodes). In the last column, we also provide the relative improvement of the root gap, thanks to the inclusion of our flow-balance constraints.

**Table 5:** Influence of the flow-balance constraints on the practical solving

Group	$(\text {SDC}_3^{\text {FB}})$		$(\text {SDC}_2^{\text {FB}})$		Rel. improvement (%)
	Root gap [%]	# Nodes	Root gap [%]	# Nodes	
SMALL	0.38	8.25	0.53	6.99	28
LARGE	9.83	0.00	11.20	0.00	12

**Table 6:** Results on dataset VIENNA (none solved to optimality)

|*K*|	*Solv* [#]		*g* [%]		*Pg* [%]	
	DLRB$_3$	B$_2$	DLRB$_3$	B$_2$	DLRB$_3$	B$_2$
5	0	0	0.77	8.33	0.00	1.88
10	0	0	0.66	6.65	0.00	1.37
20	0	0	0.88	7.33	0.00	1.10
50	0	0	1.03	22.48	0.00	0.90

The obtained results show that average gaps at the root node are significantly lower for the formulation $(\text {SDC}_3^{\text {FB}})$ and that the stronger flow-balance constraints (SDC$_3$:FB) allow for the relative improvement of lower bounds by 28 and 12% for SMALL and LARGE instances, respectively. Consequently, a larger number of instances can be solved to proven optimality within the given time limit (cf. Table 4). Concerning the comparison of lower bounds between the basic formulations $(\text {SDC}_2)$/$(\text {SDC}_3)$ and $(\text {SDC}_2^{\text {FB}})$, we did not observe any significant differences, which is why the corresponding values are left out from the table.

We conclude that the newly developed framework DLRB$_3$ scales well and provides robust performance for both small-scale and large-scale instances. We recall that the development of similar components (dual ascent, subgradient algorithm, variable reductions) for enhancing the B$_2$ approach is not straight-forward. Most of these components exploit the fact that second stage variables model an SAP in each of the scenarios, a property which does not hold in the ILP model used by B$_2$.

## Conclusions

In this article several new computational techniques for the exact and heuristic solution of the stochastic Steiner tree problem are studied. These techniques are formulated in terms of a new ILP model that is shown to be the strongest among known formulation. In the course of this study, also the previously strongest known formulation has been improved by a new class of strengthening inequalities. But perhaps most importantly, our new model enables an elegant transfer of methods known to be successful for the Steiner tree problem.

On this basis, an algorithmic framework has been designed which combines the benefits of three complementary procedures for computing lower bounds: a fast dual ascent procedure, a Lagrangian heuristic, and a Benders decomposition approach. The interaction between these methods is facilitated via an intelligent propagation of primal and dual solutions. Furthermore, these solutions are exploited in order to considerably reduce the search space via variable fixing, in which both the availability of tight bounds and problem-specific knowledge is exploited simultaneously.

An extensive computational study shows that our approach significantly outperforms state-of-the-art methods in almost all cases. The techniques presented in this work combine complementary strengths, and thus provide a rich foundation for the design of effective algorithms that perform well on a broad range of instances types. Most importantly, problem instances which can be considered as large-scale with respect to different types of properties, like the size of the instance graph or the number of scenarios, can be tackled effectively by these methods.
